# The proteome study of germinated *Puccinia triticina* urediniospores reveals a novel effector protein required for virulence

**DOI:** 10.1038/s41598-026-44996-2

**Published:** 2026-04-11

**Authors:** Ahmet Caglar Ozketen, Merve Cetinturk, Christof Rampitsch, Elifgul Aksu Tatlises, M. Burak Tatlises, Guus Bakkeren, Semra Hasancebi, Aslihan Gunel

**Affiliations:** 1 DESAM Research Institute, Near East University, Mersin 10, Nicosia, Türkiye; 2 Faculty of Arts and Sciences, Department of Chemistry, Kirsehir Ahi Evran University, 40200 Kirsehir, Türkiye; 3 Morden Research and Development Centre, Agriculture and Agrifood Canada, 101 Route 100, Morden, MB R6M 1Y5 Canada; 4 Faculty of Engineering, Department of Genetics and Bioengineering, Trakya University, Edirne, 22100 Türkiye Turkey; 5 Summerland Research and Development Centre, Agriculture and Agri-Food Canada, 4200 Hwy 97, Summerland, BC V0H 1Z0 Canada; 6 Faculty of Arts and Sciences, Department of Molecular Biology and Genetics, Kirsehir Ahi Evran University, 40200 Kirsehir, Turkey

**Keywords:** *Puccinia triticina*, Proteomics, Effectors, Leaf rust disease, Virulence, Biotechnology, Microbiology, Molecular biology, Plant sciences

## Abstract

The obligate biotrophic fungus, *Puccinia triticina* (*Pt*), is a notorious plant pathogen known for wheat leaf rust disease that devastates global crop production annually. Pathogen-host interactions need to be elucidated, especially the identification of pathogen effectors contributing to virulence. ‘Omics’ strategies are an efficient tool set to study these pathogen-host interactions. No proteome data are available on the germlings stage of *Pt*, which could highlight proteins involved in the first interaction point between *Pt *and its host. Here, we have used a proteomics approach on germinated urediniospores of *Pt* to define the germling proteome. Using two-dimensional gel electrophoresis, 167 spots in the pI range of 4–7 were observed, from which 123 proteins were identified by tandem mass spectrometry. Further in silico characterization revealed that they were predominantly involved in metabolic and cellular processes, with six proteins annotated as candidate effectors. The candidate effector PTTG_06852, hereafter named as virulence factor 1 (PtVF1), was silenced by host-induced gene silencing (HIGS) to investigate its biological function. When the expression of PtVF1 was reduced by 78%, the infection severity was suppressed by 70%. The results demonstrate a significant impairment of virulence upon silencing, which validates PtVF1 as a virulence effector of *Pt*.

**Supplementary Information:**

The online version contains supplementary material available at 10.1038/s41598-026-44996-2.

## Introduction

Wheat leaf rust is a severe disease that causes routine yield losses in all areas where wheat is grown, with much higher losses occurring during epidemics^1^. Furthermore, in warmer countries such as Turkey, rust infections can occur during any season. The causal agent, the basidiomycete fungus *Puccinia triticina* (*Pt*), is an obligate biotroph that requires a live host to complete its life cycle. Spores of *Pt*, once germinated on a wheat leaf surface, form a germ-tube which gains entry into the leaf via open stomata. From there, they colonize the apoplastic space with hyphae and eventually penetrate host cells to form intracellular feeding structures called haustoria. Nirmala and colleagues^2^ demonstrated that there is a host response even to ungerminated spores, as long as they are viable.

Plant-pathogen interactions are one of the most complex biological processes to elucidate. Through co-evolution, both plants and their pathogens have developed various elaborate mechanisms to survive and overcome pathogen attack or plant defense. A zigzag model was proposed to describe the dynamics of such plant-pathogen interactions^3^. Basal defense is activated when the plant recognizes relatively abundant pathogen-associated molecular patterns (PAMPs), such as fungal chitin, bacterial flagellin, and oomycete glucans^4^. Recognition of PAMPs by receptors triggers a defense response known as pathogen-triggered immunity (PTI). The pathogens may avoid a PTI response by deploying virulence factors (effectors) to suppress and modulate the defense response. If the PTI response is breached by the pathogen, the plant’s next level of defense relies on a specialized effector recognition system. These often exist in the form of resistance (R) proteins which are able to sense effectors or their action on other plant components. This can arrest further infection and growth of the pathogen past the infection site and is termed effector-triggered immunity (ETI). Such pathogen arrest may be caused by hypersensitive cell death (HR). In many pathosystems, the pathogen secretes effectors into apoplast, the region between plant cell wall and the plasma membrane. In the case of rusts, which form haustoria, effectors are delivered into the extrahaustorial matrix and from there into the cytoplasm. These effectors (either apoplastic or cytoplasmic) function to manipulate the host; thus, the disease progresses^5,6^. Hence, studying the proteome by focusing on secreted proteins (secretomics) allows plant pathologists to understand pathogenic virulence proteins and permits characterization of early proteins related to perception^7,8^.

Recent techniques to generate comprehensive genomes and transcriptomes allow for the extensive generation and analysis of high-throughput proteomic data^9^. There are several published reports demonstrating elevated levels of proteins at different time intervals during the disease progression of leaf rust. Song and coworkers^10^ conducted a haustoria-enriched proteome analysis to profile proteins; likewise, Rampitsch and his colleagues^11^ investigated the proteome of monoclonal antibody-purified haustoria of *Pt* race 1 BBBD. Moreover, the apoplastic proteome of *Pt* race 1 BBBD was studied extensively on resistant and susceptible wheat cultivars at different time intervals of infection^12^. At the transcriptome level, six *Pt* races were analyzed to generate an effector secretome repertoire^13^. Recently, many *Pt* effector candidates have been researched to characterize potential functions in cell death suppression. Zhang and coworkers^14^ employ transcriptome data to assay thirty effector candidates for their ability to suppress a BAX-mediated cell death response. In a similar manner, *Pt* effector candidates Pt21, Pt31812, and Pt9226 suppressed the cell death induced by BAX, whereby Pt9226 also inhibited INF1-mediated cell death in tobacco and *P. syringae* DC3000-induced cell death in wheat^15–17^. Pt3 and Pt27 produced hypersensitive cell death, suggesting avirulence functions^18^. Combining ‘omics’ analyses with *in planta* characterization experiments is crucial to discovering effector functions. However, recovery of the secreted fungal proteins from plant apoplastic fluids is complicated by their low abundance relative to host-derived proteins and cytoplasmic contaminants. Leveraging in vitro cultured fungi can mitigate some of these difficulties, even though a subset of effectors may still be missing in the absence of induction signals under plant environment. Since germlings can be produced in large quantities in vitro, it is possible to obtain a comprehensive proteome without interference from the host. For instance, Cooke and coworkers^19^ performed a proteogenomic analysis on the pathogen of pear scab disease, *Venturia pirina* to identify effector proteins by mimicking infections under in vitro conditions by tandem mass spectrometry. Similarly, germination of the urediniospores of *Pt* under environmental conditions in vitro could elucidate the proteome profile of leaf rust during the initial stage of infection.

Validation of functions for candidate genes is a necessary next step after identification. While many different methods are used for the functional analysis of candidates, RNAi-mediated gene silencing has emerged to the forefront in recent years, as gene knock-out cannot be performed in *Pt*. Host-Induced Gene Silencing (HIGS) was reported to be an efficient technique to screen fungal virulence genes^20–22^. The present study employs a proteomics approach to profile proteins of germinated urediniospores of *Pt* to understand the disease progression. We identified 123 proteins, cataloging 6 as candidate effector proteins. One of the candidates, PTTG_06852 (PtVF1), was characterized using HIGS, resulting in a significant reduction in *Pt* virulence. PtVF1, a saccharopepsin homolog, is a *Pt* effector required for full virulence in wheat to cause leaf rust disease.

## Results

### Identifying key proteins

**Fig. 1:**
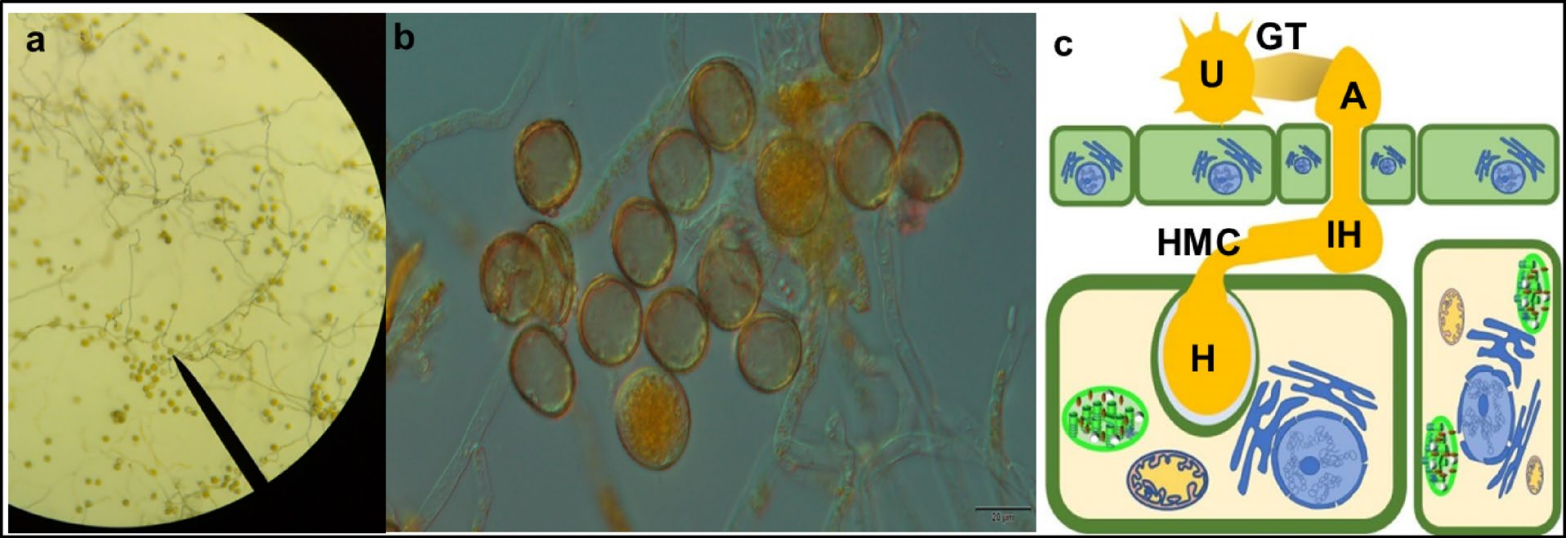
Germinated urediniospores of *Pt race 1* on 0.5% (w/v) water agar using **(a)** 40X magnification (Leica DM 500). **(b)** Olympus BX53 (40X) equipped with Olympus DP22 digi-CAM, Japan, and Axio imager 2 with NIC optics) **(c)** schematic representation of germination: U: Urediniospores, GT: Germ tubes, A: Appressorium, IH: Infection hyphae, HMC: Haustorial mother cell, and H: Haustoria. (Germination was performed at 20 °C, in the dark for 24 h in a dew chamber.)

Germinated urediniospores of *Pt* are shown in Fig. 1a. Both sides of the petri dish were examined thoroughly since some germ tubes grew towards the bottom of the dish, to avoid misidentifying ungerminated spores. Germination rates were typically close to 100%. Germinated urediniospores were photographed under a microscope (Olympus BX53) and are shown in Fig. 1b. Formation of germ tubes is visible from both angles of the petri dishes, with germination rates more than 90% (Supplementary information 1).

**Fig. 2:**
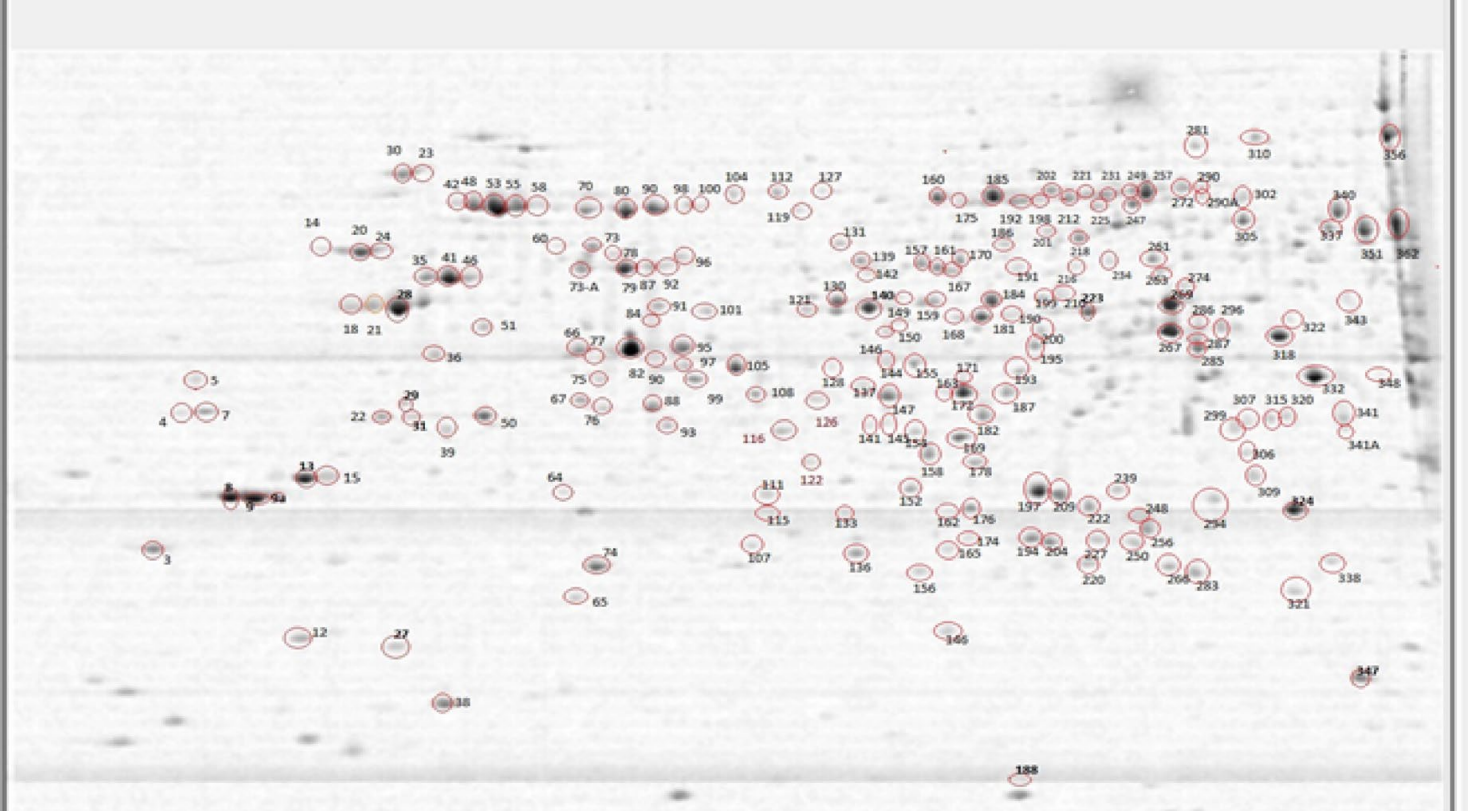
2-DE pattern (the master gel from nine gels) of germinated *Pt* race-1 urediniospores on 0.5% agarose for 24 h at 20 °C. The image was created by Proteomescan software. Gel dimensions: 24 × 20 cm. Proteins were separated with a pH 4–7 gradient in the 1st dimension, and on a 10–20% gradient SDS-PAGE in the 2nd dimension.

The 2D gels yielded, on average, 185 (± 14) well-resolved spots, of which 167 were common to at least 2 biological replicates. After excluding one known contaminant, the remaining 166 spots yielded 123 non-redundant proteins. The difference reflects repeated detection of the same proteins in multiple gel spots, a common feature of 2D-based proteomic analyses. All reported proteins represent unique accession entries. The gel image exhibiting analyzed spots is given in Fig. 2; the gel photos of all biological and technical replicates, along with image analysis, are presented in supplementary Figure S1. A complete list of identified proteins is presented in Table S1, and detailed information on proteins identified in gel spots is given in Table S1 within Supplementary Information 1. Among the 123 non-redundant proteins identified, 86% (*n* = 106) corresponded to PTTG entries, 8% (*n* = 10) to PSTG entries, and 6% (*n* = 7) to PGTG entries with the False Discovery Rate set to 1%.

### in silico characterizations

**Fig. 3:**
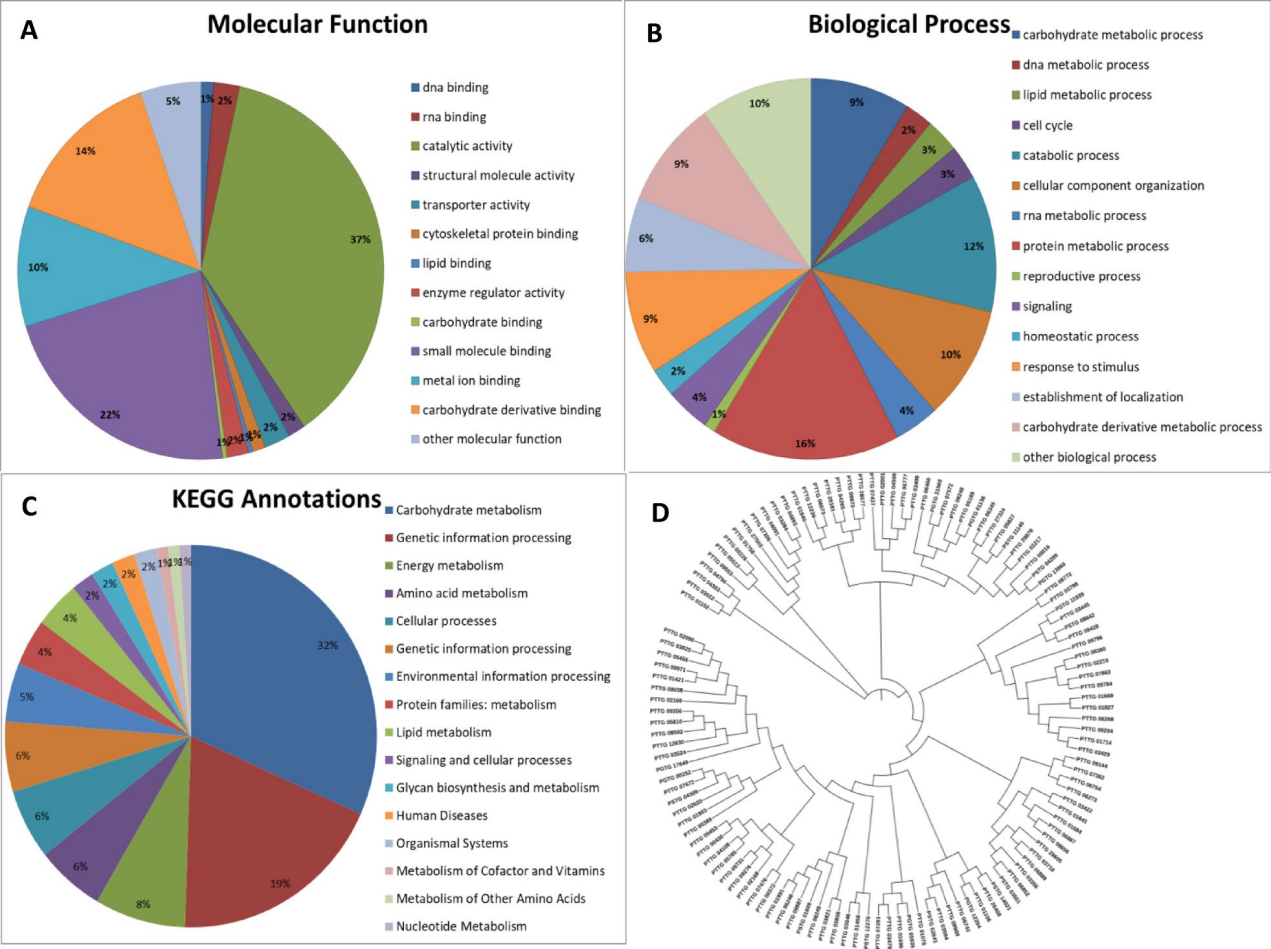
Graphical summary of in silico characterization results of the proteome data of Pt germlings in this study. Distributions of Gene Ontology (GO) annotations of **(A)** Molecular functions, **(B)** Biological processes, and **(C)** KEGG orthology (KO) analysis. **(D)** Phylogenetic tree of all identified proteins.

To understand the role of identified proteins in disease formation and progress, we applied a previously defined pipeline^23,24^. Blast2GO and BlastKOALA tools were used to annotate all proteins identified in this study (Supplementary Table 2). The graphical summary of the results is presented in Fig. 3A and B for Blast2GO and Fig. 3C for BlastKOALA. Additionally, a phylogenetic tree (Fig. 3D) was constructed from multiple sequence alignments among all identified proteins, revealing that diverse sets of proteins are present in the germlings stage. As the urediniospore transforms from a dormant stage to germination, all metabolic pathways are expected to be triggered. We observed proteins annotated in a broad spectrum of activities in terms of biological processes and molecular function. Although 38 proteins are annotated as hypothetical or uncharacterized proteins, conserved domain analysis and GO annotations generated returns for them. Consequently, all proteins have at least one annotation to evaluate their possible functions.

All proteins were analyzed for their potential role in virulence by querying them against the PHI-Base database, which houses curated known virulence factors from many pathogenic microbial species. A large number (85) of our identified proteins produced significant similarities to at least one known protein in PHI-Base (Supplementary Table 2). The 20 proteins that produced perfect hits (E-value = 0) are listed in Table 1 with their corresponding virulence attributes. These results reveal a list of conserved proteins with metabolic functions crucial for pathogenesis in different organisms.

**Table 1:** The list of 20 proteins with the best hit score in the PHI-base, and their in silico characterization results based on GO annotations and conserved domain architectures.

ID	Description	Enzymatic Action	PHI-Base ID	Conserved Domains
PGTG_12204	Beta-tubulin	Nucleoside-triphosphate phosphatase	PHI:125,079: Unaffected Pathogenicity (*A. alternate*)	PLN00220 superfamily
PSTG_03501	Actin	Hydrolases	PHI:10,434: Reduced Virulence (*B. cinerea*)	ASKHA_ATPase-like superfamily
PSTG_14021	V-type proton ATPase catalytic subunit A	Ligases; catalysing the translocation of inorganic cations; Nucleoside-triphosphate phosphatase	PHI:3321: Loss of Pathogenicity (*C. albicans*)	V-ATPase_V1_A superfamily
PTTG_00389	Cross-pathway control WD-repeat protein cpc2		PHI:4182: Reduced Virulence (*C. neoformans*)	WD40 Superfamily
PTTG_01106	Tubulin alpha-1 A chain	Hydrolases	PHI:2530: Loss of Pathogenicity (*A. fumigatus*)	Tubulin_FtsZ_Cetz-like superfamily
PTTG_01152	Uncharacterized protein PtA15_10A368	Oxidoreductases	PHI:124,492: Reduced Virulence (*F. graminearum*)	HemY superfamily
PTTG_01458	Phosphoenolpyruvate carboxykinase [ATP]	Phosphoenolpyruvate carboxykinase (ATP); Transferring phosphorus-containing groups	PHI:424: Reduced Virulence (*C. neoformans*)	PEPCK_HprK superfamily
PTTG_03046	Phosphoenolpyruvate carboxykinase [ATP]	Phosphoenolpyruvate carboxykinase (ATP); Transferring phosphorus-containing groups	PHI:424: Reduced Virulence (*C. neoformans*)	PEPCK_HprK superfamily
PTTG_03356	Glucose-6-phosphate isomerase	Glucose-6-phosphate isomerase	PHI:9310: Reduced Virulence (*E. amylovora*)	pgi superfamily
PTTG_03422	6-phosphogluconate dehydrogenase, decarboxylating 1	Phosphogluconate dehydrogenase (NADP(+)-dependent, decarboxylating)	PHI:9835: Reduced Virulence (*B. cinerea*)	Gnd superfamily
PTTG_03594	Mitogen-activated protein kinase 1	Mitogen-activated protein kinase	PHI:124,893: Increased Virulence (*U. esculenta*)	PKc_like superfamily
PTTG_03821	S-adenosyl-L-homocysteine hydrolase		PHI:11,601: Reduced Virulence (*F. graminearum*)	AdoHcyase superfamily
PTTG_04108	Fumarase fum1	Fumarate hydratase	PHI:10,048: Reduced Virulence (*S. enterica*)	FumC superfamily
PTTG_05785	Succinate dehydrogenase [ubiquinone] flavoprotein subunit, mitochondrial	Succinate dehydrogenase (quinone)	PHI:4966: Unaffected Pathogenicity (*Z. tritici*)	FAD_binding_2 superfamily
PTTG_05827	Heat shock protein 60	Nucleoside-triphosphate phosphatase	PHI:3085: Increased Virulence (*P. gingivalis*)	chaperonin_like superfamily
PTTG_06777	Thioredoxin-disulfide reductase	Thioredoxin-disulfide reductase; Protein-disulfide reductase	PHI:2846: Lethal (*C. neoformans*)	TRX_reduct superfamily
PTTG_06867	Heat shock protein 90	Nucleoside-triphosphate phosphatase	PHI:12,133: Reduced Virulence, Unaffected Pathogenicity (*C. neoformans*)	HSP90 superfamily
PTTG_08360	Pyruvate kinase	Pyruvate kinase	PHI:8611: Unaffected Pathogenicity, Reduced Virulence, Loss of Pathogenicity (M. oryzae)	Pyruvate_Kinase superfamily
PTTG_26408	V-type ATPase		PHI:3321: Loss of Pathogenicity (C. albicans)	V-ATPase_V1_A superfamily
PTTG_29181	Spermidine synthase		PHI:2239: Reduced Virulence (*U. maydis*)	AdoMet_Mtases, PLN02819 superfamily

Next, we looked for signal peptides in our identified protein, which would indicate that they may be secreted. Only six proteins were predicted as possessing a signal peptide, and three of these were predicted to be cytoplasmic effectors via EffectorP v3.0 (Table 2). The mature protein sequences (without signal peptide region) were subjected to subcellular localization analysis using different tools. The predictions reveal endoplasmic reticulum localizations for all of the proteins (Table 2). Two of the candidates (PTTG_06852 and PTTG_00016) were annotated with protease functions, whereas PTTG_06852 also gave a significant hit to the known ugt51E1 (an aspartic protease, Uniprot ID: Q8NJS2) gene of the canola blackleg fungus, *Leptosphaeria maculans*^25^. PTTG_06852 is also homologous (E-value: 6.02 × 10^− 155^) to AaPep4 (A0A177D640) of *Alternaria alternata* (rot fungus). AaPep4 is also a vacuolar protease^26^. Vacuolar proteases are known for their contribution to autophagy and virulence processes^27^. Annotations of the six effector candidates are presented in Table 2.

**Table 2:** The list of predicted secreted effector candidates and their in silico annotations.

SeqName	Description	Enzyme Names	Conserved Domains	PHI-Base ID	DeepLoc 2.1
PTTG_06852 (PtVF1)	Vacuolar protease A	Acting on peptide bonds (peptidases)	Pepsin_retropepsin_like superfamily	PHI:697: Unaffected Pathogenicity (L. maculans)	N, M, E.R.*
PTTG_01827	Protein disulfide-isomerase precursor	Protein disulfide-isomerase	ER_PDI_fam superfamily	PHI:9867: Reduced Virulence, Unaffected Pathogenicity (U. maydis)	N, M, E.R.
PTTG_00016	Serine protease	Acting on peptide bonds (peptidases)	Peptidases_S8_S53, Inhibitor_I9 superfamily	PHI:9903: Reduced Virulence (B. cinerea)	N, M, E.R.
PTTG_07281	-	-	PTZ00009 superfamily	PHI:123,424: Unaffected Pathogenicity (M. oryzae)	N, M, E.R.
PTTG_02001	Hypothetical protein	Exopoly-phosphatase	DHH, DHHA2 superfamily	-	N, M, E.R.
PTTG_09276	Proteophosphoglycan ppg4	-	Ish1, TrbL superfamily	-	C, E.R.

*Subcellular localization predictions of candidate effectors were conducted using mature protein sequence (i.e., without signal peptide). N: Nucleus, M: Mitochondria, E.R.: Endoplasmic Reticulum, C: Cytoplasm.

**Fig. 4:**
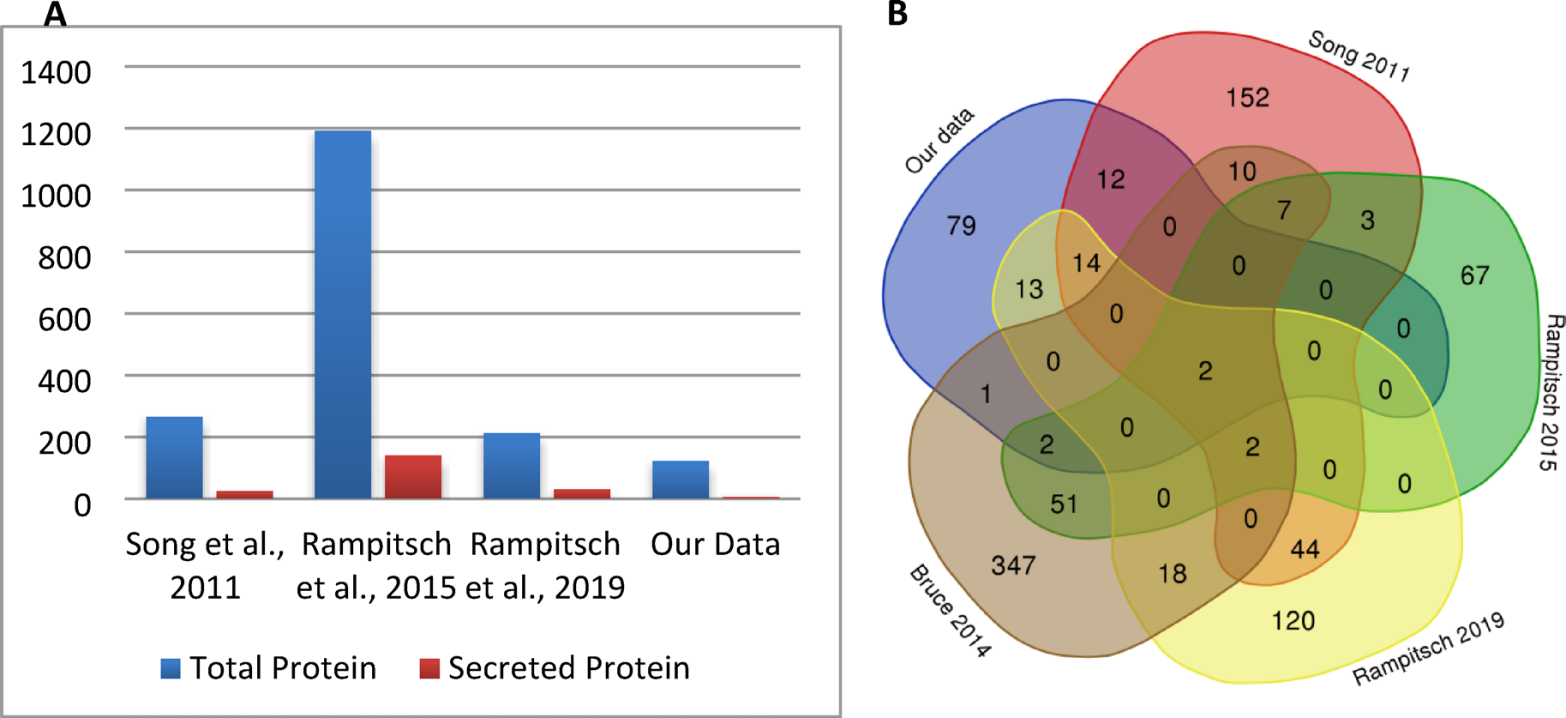
**(A)** Comparison of the number of identified secreted proteins over the total number of proteins in other *Pt* proteome studies, **(B)** Schematic representation of overlapping proteins identified in other reports.

In order to understand the novelty of the discovered proteins, we conducted a comparative analysis against published works on *Pt* where various sets of proteins or genes were revealed as present or induced during different stages of infection^10–13^. The number of identified secreted proteins versus all proteins is compared with our data in Fig. 4a. The overlapping proteins in the previous studies were identified and represented as a Venn diagram in Fig. 4b. The comparison shows that 79 of 123 proteins identified in our data were specific to this study, designated with blue color in Fig. 4b. Four effector candidates were also reported in the other works, whereas PTTG_09276 and PTTG_02001 are newly reported in this work.

### Investigating the virulence function of candidate effector PtVF1

**Fig. 5:**
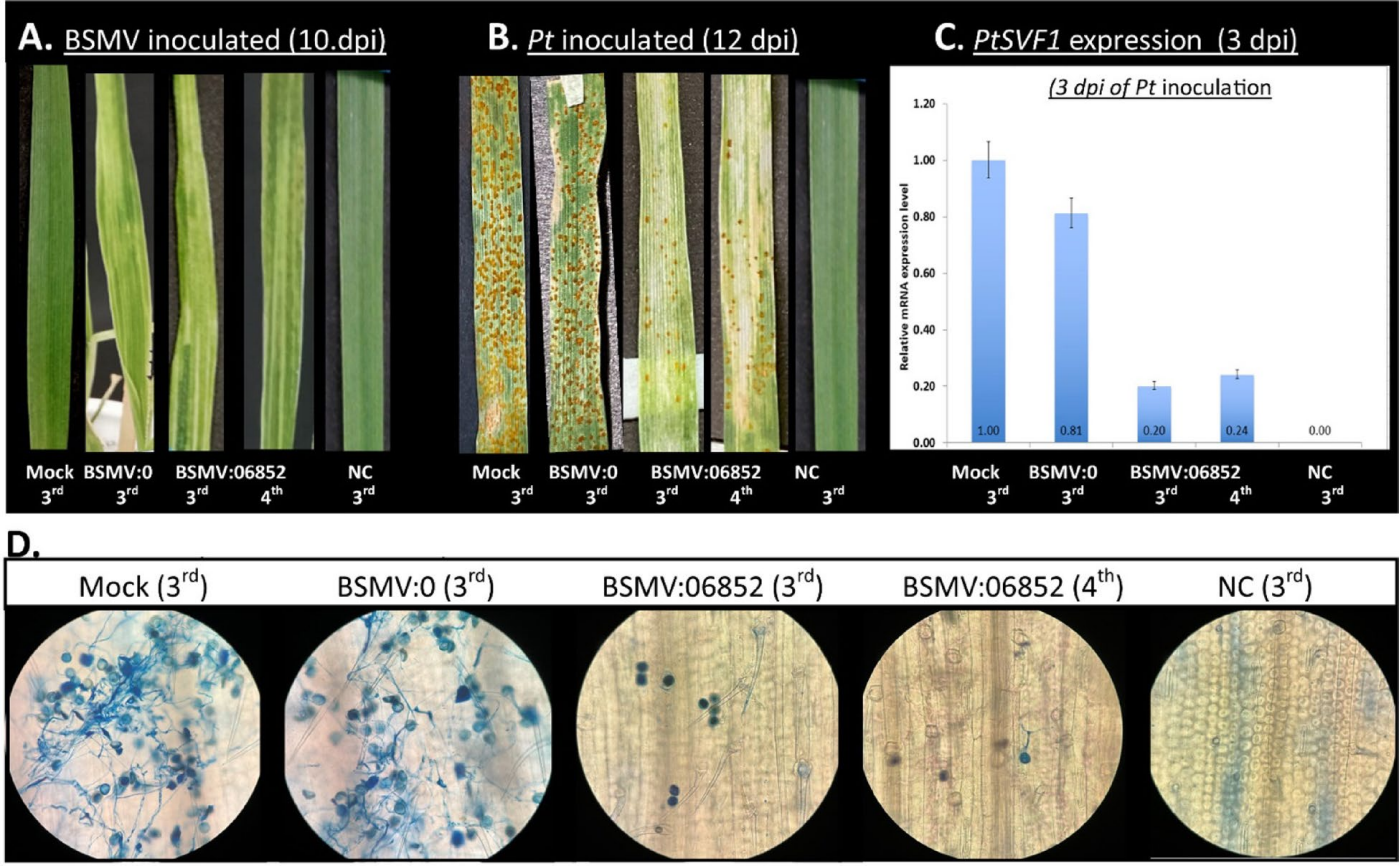
Functional analysis of PtVF1 via BSMV-HIGS assay **(A)** Viral symptoms at 10 dpi of the BSMV (**B)** *Pt* infection results at 12 dpi of *Pt* inoculation **(C)** Expression level of the PtVF1 at 3 dpi of *Pt* inoculation. Data were normalized to the *Pt* Succinate dehydrogenase gene. Error bars indicate standard deviation (SD). Statistical significance was determined using a two-tailed Student’s t-test (*p* < 0.05). **(D)** Images of the *Pt* hyphal development under the microscope at 3 dpi of *Pt* inoculation. (**Mock**: Leaves treated with 1X FES buffer, **NC**: Leaves without any treatment, **3rd** : Third leaf, **4th** : Fourthleaf)

One of the identified effector candidates, PTTG_06852, was annotated as a secreted vacuolar protease. Without its signal peptide sequence, PTTG_06852 was predicted to reside in the host nucleocytoplasmic or mitochondrial localization. PTTG_06852 was calculated to be 46.72 kDa in size and to be enriched in cysteine (five residues). The vacuolar proteases of phytopathogenic fungi are described to function in autophagy and pathogenicity-related pathways, making PTTG_06852 a promising candidate for virulence^27^. Therefore, the potential function of PTTG_06852 was tested by transient knockdown using HIGS. A region matching specifically identified peptides was selected as a target, and a complementary RNAi fragment (495 bp) was designed for this region. The fragment was cloned in sense (pϒ:06852s) and antisense (pϒ:06852as) orientations, and a mixture of their run-off viral transcripts was used together as BSMV inoculation. The second leaves of the plant (2-leaf stage) were inoculated. We monitored the silencing symptoms on the 3rd and 4th leaves after they appeared. 80% of the BSMV-inoculated plants exhibited viral symptoms by white-yellow stripes on the 3rd and 4th leaves at 10 dpi (Fig. 5A). Additionally, a bleaching phenotype was observed on the 3rd and 4th leaves of the BSMV: PDS control group, which serves as evidence of dsRNAs delivery to new cells and effective gene silencing in this RNAi experiment. At this stage, the same seedlings were inoculated with *Pt* race 1 urediniospores. The dramatic reduction of the *PTTG_06852* gene expression level was demonstrated by qRT-PCR at 3 days after *Pt* inoculation. There was an average reduction of 78% in the expression level of the gene in the BSMV:06852 group plants compared to plants inoculated with the BSMV:0 control (Fig. 5C). The infection severity was assessed on the 0 to 100% modified Cobb scale (Fig. 4D), and fungal biomass was evaluated by qRT-PCR. In the samples with reduced *PTTG_06852* gene expression, it was observed that *Pt* infection had significantly decreased compared to BSMV:0 at 12 dpi. The infection severity was 20% and 30% at the 3rd and 4th leaves, respectively, with a biomass reduction of approximately 77% and 61% measured by qRT-PCR. These results demonstrate that PTTG_06852 is indeed required for virulence; hence, we named it PtVF1. Despite the reduction in infection, rust pustules would still appear, but these were notably much smaller compared to the controls (Fig. 5B). Microscopic examination also revealed significant retardation in hyphal development from urediniospores on the 3rd and 4th leaves (Fig. 5D). Hyphal lengths are significantly (Kruskal-Wallis test, *p* < 0.001) impaired subsequent to PtVF1 silencing on both 3rd and 4th leaves of wheat compared to the control group (Supplementary Figure 5). The results from the silencing experiment and the in silico prediction suggest that PtVF1 could play a role during the early stages of the infection. PtVF1 could function in the cytoplasm as a cytoplasmic effector if it translocates into the host cell. Therefore, future studies are required to elucidate how PtVF1 contributes to the virulence.

## Discussion

Proteins from *Pt* germlings were first separated by 2-DE in 1982^28^, showing a relatively simple proteome that did not overwhelm the resolving power of the gels. At that time, protein identification from 2D gels was not possible. The proteome of *Pt* was previously studied, specifically focusing on haustoria or apoplastic fluid^10–12^. The studies pointed out a list of proteins involved in a broad spectrum of metabolic pathways, along with a subset of secreted proteins. The percentage of secreted proteins compared to total identified proteins was around 10% for these studies. None of these works aimed to screen germinated urediniospores for leaf rust. Zhao and colleagues^29^ recently compared the proteomes of ungerminated and germinating spores of *P. striiformis* (stripe rust) using iTRAQ labels and an LC-MS approach. They identified 1548 proteins, of which 64 increased, and 54 decreased in abundance. Here, we have conducted a similarity analysis against known virulence factors in PHI-Base to investigate the potential function of the identified proteins in our study. Up to 70% of the proteins showed significant similarity to known factors, while some of them matched with more than one entry (Supplementary Table 2). Twenty of the proteins listed in Table 1 are nearly identical (e-value: 0) to the known virulence factors they matched. We compare the results with other published works to relate shared and unique proteins between each research (Fig. 4). A large proportion of the proteins (79) are unique to our research, whereas the remaining proteins overlap with the proteins reported to be present in different structures (haustoria, etc.) and time intervals of *Pt*.

In this study, we have generated the proteome data of germinated urediniospores by identifying 123 proteins with functional annotations in various metabolic processes. This result was anticipated since urediniospores alter their dormant stage through germination to establish a biotrophic interaction. Proteins involved in catabolism, energy production, and transport were found to be especially enriched in germlings, indicative of the needs of the germinated spores. We have also identified 19 oxidoreductases (15%) and 31 hydrolases (25%), which could function to manipulate self and host factors^30^. Hydrolases are abundant in fungal secretomes, such as cellulase, xylanase, pectinase, chitinase, etc., and they are known for their involvement in the infection process^31–33^. Seven of the hydrolases belong to the protease/peptidase subgroup. Among them, we have three proteasome complex-related peptidases, a vacuolar protease, and a serine protease with predicted secretion signals that show significant homology to known virulence factors in PHI-Base, while the remaining two (a mitochondrial peptidase and a hypothetical protein with peptidase activity) proteins do not. The proteases from fungi are known to contribute to virulence through various mechanisms, including cell wall degradation, cellular response, etc.^34–36^. Moreover, these proteases may have extracellular functions to directly interact with the host factors or interfaces such as chitinase-modifying protein from *Bipolaris zeicola*, fungalysin metalloprotease from *F. verticillioides*, and serine protease from *Verticillium dahliae*^37–39^. This high percentage of hydrolases, especially proteases, reported in this study underlines their importance in the germination stage. Possible functions in virulence of these proteases need further validation studies.

In addition to hydrolases, fungal oxidoreductases have a role in adjusting host-linked oxidative stress^40^. There are several reports demonstrating the significance of oxidoreductases for a broad range of plant fungal pathogens^41–43^. The presence of oxidoreductases is crucial to counter oxidative stress response by typically neutralizing reactive oxygen species (ROS) of the host plant^40^. An elevated level of ROS could impair the development/growth of the fungus by endangering the integrity of proteins, lipids, and DNA. The number of annotated proteins with these functions in our data demonstrates germination is a central step to ensure successive phytopathogenicity (Supplementary Table 2). The identified oxidoreductases may be implicated in virulence by fine-tuning the ROS levels to maintain cellular redox homeostasis of the fungus in the hostile environment of the host. For comparisons, we used the PHI-Base collection of pathogenicity factors and revealed that 18 of 19 proteins annotated as oxidoreductases have similarity to known virulence factors.

Our proteomic profiling shows the remaining annotated proteins belong to different enzymatic activity groups of transferases (17), lyases (14), isomerases (12), ligases (5), and translocases (3). Glutamine synthetase (GS), which is one of the identified ligase proteins, coordinates cellular nitrogen pools for the development of the germ tube and the primary infection structure^44^. Glutamine accumulates in urediniospores of *Puccinia helianthi*, functioning in nitrogen storage and biosynthesis^45^. Collectively, the reports from obligate parasites and filamentous pathogens strongly suggest that GS in *Pt* is not merely a housekeeping enzyme but a key regulatory factor and metabolic nexus linking nitrogen assimilation, developmental regulation, and successful host penetration^46–48^. Similarly, another ligase, Glutathione Synthetase, was identified in the *Pt* germ tube. Glutathione (GSH) is critical for maintaining cellular redox balance and adjusting pathogen-induced ROS levels^49,50^. Indeed, the close link between GSH concentration and morphological transitions, such as germ tube formation, has been previously established in other major fungal pathogens, such as *Magnaporthe oryzae*^46^ and *Puccinia* species, where antioxidant pathway genes are highly expressed during urediniospore development^45,48^. Taken together, our finding places GS as a core regulatory enzyme in *Puccinia* for managing oxidative stress and the host penetration program.

We labeled 17 proteins with transferase activities involved in energy and carbohydrate metabolism. The detection of these metabolic enzymes in the *Pt* germ-tube proteome indicates a strong activation of central carbon metabolism during the germination stage. Although our observation is in the absence of the host environment, it could still give clues about the proteins associated with early infection development. These enzymes collectively support ATP production and biosynthetic precursor supply, as previously noted in infection structures of *Pt* and other fungi^10,51,52^. Moreover, several metabolic enzymes may exert secondary moonlighting functions in adhesion or host interaction^53^. The identified mannose-1-phosphate guanyltransferase (GMPP), UDP-N-acetylglucosamine pyrophosphorylase (UAP), and UTP-glucose-1-phosphate uridylyltransferase (UGPase) in *Pt* germ tubes indicate a redirection of carbon metabolism toward cell-wall and biosynthesis for host penetration. These enzymes are essential for the formation of chitin, β-glucans, and mannoproteins, supporting rapid infection-structure development^10,54^, and these findings have been reported in other phytopathogens^55,56^. Thus, their enrichment in our dataset likely reflects a metabolic pre-adaptation for host invasion.

Kinases play critical roles in regulating various infection processes, particularly in pathogenic fungi. Mitogen-activated protein kinase 1 (MAPK1), a previously reported protein to contribute to virulence, is one of these kinases^21,57,58^. In the wheat stripe rust pathogen *Puccinia striiformis* f. sp. *tritici* (Pst), PsMAPK1, a YERK1 MAP kinase belonging to the Fus3/Kss1 class, has been identified^59^. As direct evidence of this cascade’s function, treatment of *Pst* urediniospores with the STE11 MAPK kinase activation inhibitor resulted in the production of deformed germ tubes, demonstrating that the MAPK cascade plays an important role in germ tube morphology and early development^60^. Furthermore, phosphoproteomic analyses have revealed that MAPK signalling pathways are significantly enriched in the incompatible interaction exhibited by wheat against *Pst* during the early stages of infection^59^. Calcium/calmodulin-dependent kinases (CaMKs) are Ser/Thr protein kinases that respond to changes in cytosolic free Ca²⁺ and perform various roles in eukaryotes. PsCaMKL1, a CaMK-like protein kinase specific to Basidiomycetes, appears to be essential for the full virulence of *Pst* and is thought to play a role in the invasive growth of the pathogen^61^. In general, protein kinases (PKs) are evolutionarily conserved in plant pathogenic fungi, as in other eukaryotes, and play major regulatory roles. It has been demonstrated that most PKs are important for pathogenesis in model fungal pathogens^62^. This situation suggests that the kinases found in the *Pt* germ tube proteome are part of the fundamental mechanisms for fungal virulence.

We detected 14 proteins annotated as lyases, such as isocitrate lyase (ICL), enolase, fumarase, fructose bisphosphate aldolase (FBA), and phosphoenolpyruvate carboxykinase (PEPCK), suggesting a unique metabolic flexibility mechanism that is critical for the pathogen’s successful establishment in the host tissue. ICL is the key enzyme in the glycine cycle that promotes growth on C2 compounds such as fatty acids, and this metabolic pathway is essential for the survival of many plant and human pathogens within their hosts^63^. *Pst* has the inactive form of the ICL gene (PstICL1), highly expressed in urediniospores, and the inhibition of ICL significantly reduces spore germination rates^64^. In parallel, the temporal regulation of the pre-penetration phase of *Magnaporthe grisea* infection relies on ICL-dependent glyoxylate cycle^65^. Another crucial enzyme in this class is fumarase, involved in energy metabolism and DNA repair networks, highlighting the metabolic basis of virulence in pathogenic fungi. For instance, the reduction in growth, stress tolerance, and virulence has been observed upon FpFumB loss in *Fusarium proliferatum*^66^, and the maintenance of cellular energy flow by fumarase activity present in both soluble and particulate fractions in *Fusarium oxysporum*^67^. In our study, PEPCK, fructose biphosphate aldolase, and enolase, related to gluconeogenesis and glycolysis, are detected. In plant pathogenic fungi, glycolysis–gluconeogenesis enzymes that regulate energy and carbon flux during germ tube formation are typically activated. Species such as *Phakopsora pachyrhizi*^68^ and *Colletotrichum acutatum*^69^ show an increase in these metabolic enzymes during the early infection stage, which is consistent with the enzymes observed in our *Pt* germ tube proteome data. This suggests that this metabolic shift is an evolutionarily conserved strategy in preparation for host entry^70^.

The isomerases and mutases, such as protein disulfide-isomerase, mannose-6-phosphate isomerase, peptidylprolyl isomerase, etc. (Supplementary Table 2), detected in the germ tube stage may contribute to the metabolic flexibility of *Pt* by playing a role in protein folding, carbon metabolism, and cell wall biosynthesis. It has been demonstrated in various plant pathogens that FK506-binding protein (FKBP/PPIase) and peptidyl-prolyl cis-trans isomerases are critical for the proper folding/secretion of virulence factors and for the effector maturation of protein disulfide isomerase (PDI) in the ER; disruption of these processes impairs development and virulence^71^. Additionally, loss of function in carbon flux/cell wall precursor enzymes such as phosphomannose isomerase (PMI), phosphomannomutase (PMM)/hexosamine pathway components, and glucose-6-phosphate isomerase (GPI) disrupts growth-cell wall integrity and reduces virulence, providing a framework that explains the energy and structural requirements for rapid germ tube growth and penetration^72^.

A subset of effectors plays a significant role in the early stages of infection and hence in overall virulence. They can suppress host defense and interact with the host cellular signals to regulate the host cell metabolism, including transcription, to develop a successful infection^73^. At the beginning of the attack, fungi synthesize effector proteins and secrete them to the host cells for defense suppression and regulation^74,75^. The first group of effectors thus acts to create favorable conditions during the formation of infectious hyphae. In the middle phase of the attack, another group of effectors acts to facilitate metabolic exchange, such as nutrients, transcription factors, and some metabolites. Another group of effectors plays a role in the late-stage attack, hijacking the host’s transcriptional and translational machinery to suppress host defenses and to reorganize the host’s cellular machinery to support completion of the pathogen life cycle. Some of the effectors target mitochondria and chloroplasts to control energy^75–77^. Identifying all of the rust effector proteins is crucial for developing a powerful and effective management strategy against the pathogen. We profiled the proteins involved in germination and dissected the candidate secreted effectors by using a well-studied prediction pipeline. Six effector candidates were predicted to possess a known signal peptide using SignalP 6.0^78^ (Table 2). One of these, PTTG_01872, was annotated as protein disulfide isomerase (PDI). Several studies demonstrate that PDI plays an important role in pathogenicity. A study with *Fusarium graminarum* demonstrated that FgEps1 plays a role in the formation of disulfide bonds in secreted proteins, and it was observed that these bonds significantly increase the virulence of the pathogen^76^. A study with *Botrytis cinerea* demonstrated that BcPDI1 is involved in protein folding, redox homeostasis, and NADPH oxidase signaling, which are vital for the fungus, and reported to play a key role in fungal development and infection^79^. PTTG_00016, another identified effector candidate, was annotated as a Peptidase S8/S53 domain-containing protein that has a serine-type endopeptidase activity localized in the extracellular domain. Serine proteases containing this domain have been implicated in plant-fungus interactions, including host tissue degradation and manipulation of plant defense mechanisms^80^. Furthermore, genomic analyses have revealed an expansion of serine protease genes, including the S8 and S53 families, in various phytopathogenic fungi^81^. No studies are available for the remaining effector candidates, PTTG_07281, PTTG_02001, and PTTG_09276, in terms of annotated biological functions.

PtVF1 (PTTG_06852) is predicted to have a protease function and matches a known virulence factor in PHI-Base, and was considered a strong effector candidate. Consequently, we chose PtVF1 for further characterization as a candidate effector for functional validation. PtVF1 was predicted to target the ER, but it also bore a nuclear localization signal and a mitochondrial transit peptide region (Table 2). To be able to establish a successful infection, the invading hyphae must rapidly find a source of energy to support their growth. Chloroplasts and mitochondria, as the main energy-providing organelles of the host cells, are logical targets for pathogen proteins. It was reported that pathogen effectors can mimic host transit peptide sequence to target these organelles, although the functions of these proteins are yet to be discovered^82–84^. Both mitochondria and chloroplasts produce reactive oxygen species (ROS) that are important for host defense, and it could be speculated that some of the effectors targeting these organelles could interfere with ROS production and signaling, to manipulate biological processes ongoing in these organelles. PtVF1 possesses more than 5 cysteine residues, which is an additional feature of known effector proteins^23,85,86^. When we searched the literature for other aspartic proteases similar to the PtVF1 protein sequence, we found that a soybean rust effector (PpEC15) from *Phakopsora pachyrhizi* is a homolog of PtVF1. PpEC15 suppresses PAMP-induced ROS production and enhances bacterial growth while interacting with a peptide-chain release factor (PCRF), an NAC83 (NAM, ATAF, and CUC) transcription factor, and a DAHP (3-deoxy-7-phosphoheptulonate) synthase^87^. In that study, it was also shown that PpEC15 can cleave DAHP but does not cleave PCRF or NAC83 of the host. Subsequently, we evaluated the predicted interacting partners of PtVF1 using the String database v12.0. The results reveal that all of the predicted partners contain peptidase domains (Supplementary Fig. 6a). When we repeated the analysis with a host homolog of PtVF1, an aspartic protease of wheat (A0A3B5Z4G2), and similarly, the interacting partners are peptidases forecasting the target proteins of PtVF1 in the host (Supplementary Fig. 6b).

Our HIGS results to validate a potential function of PtVF1 in virulence showed that the observed reduction of the infection development was directly correlated to the rate of silencing of the *PtVF1* gene, resulting in the near-complete halt of hyphal development (Fig. 5). The suppression of the development of infection structures when the gene was silenced demonstrated that PtVF1 was highly effective at the early stages of *Pt* infection and has a function in virulence, a hallmark of a true effector. One of the important functions of virulence factors is to activate the formation of the main infection structures, such as haustoria. The contribution of PtVF1 to virulence could be related to structural impairment in the host. Plant pathogenic fungi/oomycetes employ proteases to break down host barriers and to activate developmental signals for haustorium formation^89^. A subtilisin-like protease of the hemiparasitic *Phtheirospermum japonicum* fungus (PjSBT1.2.3) drives the process and activation of an endogenous peptide hormone, CLAVATA3/Embryo Surrounding Region 1 (PjCLE1), responsible for haustoria development^90^.

## Conclusion

Effectors are referred to as virulence factors of pathogens that play active roles in the development of the infection. Comprehending the leaf rust disease is possible by mapping these virulence factors and elucidating the host-pathogen interaction. Research on rust pathogens is complicated by the fact that it is an obligate biotrophic parasite, and that it cannot be genetically modified to produce knock-outs. However, germlings can be grown in vitro, and subsequent analyses are therefore not hampered by the presence of the host proteome. To the best of our knowledge, we have described the germlings’ proteome profile of *Pt* here for the first time to pinpoint candidate effector proteins and virulence factors. Among the identified candidate effectors, we demonstrated that PtVF1 is an effector contributing to the virulence. Utilizing an omics strategy in the research of plant fungal pathogens is a promising tool assisting future applications in effector-mediated breeding and plant-pathogen interactions.

## Materials and methods

### Fungal strain and plant material

*Puccinia triticina* Eriks race-1 (BBBD) urediniospores were maintained on hexaploid bread wheat (*Triticum aestivum* L.) cv ‘Morocco’ in a growth chamber under 16 h/8 h day/night cycle at 20℃/15℃, respectively. Urediniospores were collected from pustules and germinated on 0.5% (w/v) agarose in water for 24 h at 20℃ in the dark.

### Protein extraction from germlings

Germlings were observed under a microscope (Leica DM500). Plates containing dense germling mats (90–95% germinated) were used in this study. A total of 300 mg of urediniospore was used in each biological replicate in a total of three biological replicates. Protein was extracted from germlings using a combination of acetone-TCA and phenol-SDS extraction^91^. Germlings on agarose were ground in liquid N_2_ and transferred to 10 mL cold acetone containing 10% (w/v) TCA and 0.07% (w/v) DTT and left overnight to precipitate at -20 ℃. Samples were then centrifuged at 16,000 *g* for 10 min at + 4 ℃. The precipitate was washed 3 times with cold methanol containing 0.1 M ammonium acetate. The final pellet was washed with cold acetone containing 0.07% (w/v) DTT, dried, and weighed.

The acetone powder was resuspended with vortexing at 4 °C for 5 min in 0.6 ml of SDS buffer (30% (w/v) sucrose, 2% (w/v) SDS, 0.1 M Tris-Cl, 0.2% (w/v) DTT, 1 mM PMSF, pH 8.0) and mixed 1:1 with Tris-buffered phenol (pH 7.6) per 100 mg powder. The resulting suspension was centrifuged at 16,000 *g* for 10 min at + 4℃, and the phenol phase was transferred to a new tube. The aqueous phase was re-extracted, and the two organic phases were pooled and precipitated with 4 volumes of cold 0.1 M ammonium acetate in methanol, overnight. The precipitate was collected, washed with cold absolute methanol and then with cold acetone containing 0.07% DTT, and dried. The pellet was resuspended in 7 M urea, 2 M thiourea, 4% (w/v) CHAPS, 20 mM DTT, sonicated in 6 × 15 s bursts, and centrifuged at 30,000 *g* for 30 min. The supernatant was retained and assayed for protein content using the Bradford assay with BSA standards^92^.

### Two-dimensional electrophoresis

Two-dimensional gel electrophoresis (2-DE) separations were carried out as follows: IPG strips (24 cm, pH 4–7) were rehydrated with 450 µL solution containing 700 µg of protein for each sample in a rehydration tray at 20 °C for 12 h. Rehydrated IPG electrophoresed in an IPGphor3 unit (GE Healthcare) at 20 °C. The IEF program was: 100 V for 30 min, 250 V for 250Vh, 500 V for 500Vh, 1000 V for 1500 Vh, 10 kV for 22 kVh, and 10 kV for 5 h. Then, strips were placed in an equilibration solution (6 M urea, 30% (w/v) glycerine, 2% (w/v) SDS, 50 mM Tris.HCl, pH 8.8) containing 1% DTT and 2.5% iodoacetamide, respectively, for 20 min.

For separation in the 2nd dimension, equilibrated strips were then transferred to 10–20% SDS polyacrylamide gradient slab gels. Electrophoresis was carried out at 15 °C with a 1.5 W/gel for the first hour, followed by a 20 W/gel until the bromophenol blue dye marker reached the bottom of the gel. 2-DE was performed as 3 technical replicates for each biological replicate. Gels were stained with colloidal coomassie brilliant blue (CBB) and digitalized by Syngene Proteomescan Pro 2D Gel Imaging Systems at 800 dpi, and Dymension (Syngene) was used for image analysis. Spots observed in at least two biological replicates were selected and cut from gels and reduced to cubes of approximately 1mm^3^. In-gel trypsin digestion and peptide extraction were achieved exactly as described in . Following image analysis, spots observed in at least two biological replicates were selected and cut from gels and reduced to cubes of approximately 1mm^3^. In-gel trypsin digestion and peptide extraction were achieved exactly as described in.

### Mass spectrometry analysis

Mass Spectra of the resulting peptides were acquired in a hybrid quadrupole-orbitrap mass spectrometer (Q-Exactive: ThermoFisher, Bremen, Germany). Tryptic peptides were separated through a C_18_ column (12 cm fused silica column, 75 μm ID, packed with Vydac C_18_, 3 μm beads, 300 Å pores) with nanoelectrospray ionization. An acetonitrile gradient (2% (v/v) to 40% (v/v) in 0.1% (v/v) FA) was delivered at 300 nl/min over 24 min (Easy nLC1000: ThermoFisher, San Jose, CA). A survey scan acquired over the range m/z 300–2000 was followed by 12 MS^2^ scans of the most intense ions, with dynamic exclusion set to 6 s. Protein identification of the MS^2^ spectra was performed using Mascot Server v2.4 (MatrixScience, London, UK). The following parameters were set: a monoisotopic mass accuracy of ± 5 ppm; up to one missed cleavage; peptide charge up to + 5; fixed modification of carbamidomethyl (C), and variable modifications of oxidation (M) and deamidation (NQ). Raw MS data files were converted to MGF using Mascot Distiller (v2.5.1: MatrixScience) and used to query a database of the genomic sequences of *P. triticina* strain 1–1 BBBD Race 1 (GCA_000151525.2) , *P. striiformis* f. sp. *tritici (Pst)* strain PST-78 (GCA_001191645)^94^, and *P. graminis* f. sp. *Tritici (Ptg)* strain CRL 75-36-700-3 (GCA_000149925.1)  containing 52,198 sequences downloaded from the Broad Institute Cambridge MA, (https://www.broadinstitute.org/scientific-community/science/projects/fungal-genome-initiative/). The sequences are now available on the EnsemblFungi database (https://fungi.ensembl.org/). The *Pt*, *Pst*, and *Pgt* databases (the accession header: PTTG = *P. triticina*, PSTG = *P. striiformis*, PGTG = *P. graminis*) were combined into one for convenience; all of these rust species infect wheat and have well-annotated genomes. Of the identified proteins, 86% (*n* = 106) corresponded to *Pt* entries, 8% (*n* = 10) to *Pst* entries, and 6% (*n* = 7) to *Pgt* entries, with the False Discovery Rate set to 1%. Proteins were considered correctly identified if returns contained two or more peptides with a significant ion score as defined in . The mass spectrometry proteomics data have been deposited to the ProteomeXchange Consortium via the PRIDE partner repository with the dataset identifier PXD007162.

### Data analysis

Gene ontology annotations were generated using the BLAST2GO tool^97^(www.blast2go.org) by querying with BLASTp against the nonredundant (nr) database of NCBI (August 2025) and annotating at an E value of ≤ 1.0 × 10^− 3^. BlastKOALA was used to characterize KEGG orthology (KO) annotations of the proteome data with default parameters searching eukaryotic reference genes^98^. Annotation was based on the NCBI description lines from the BLASTp return with the smallest E-value. Multiple sequence alignment of identified proteins was analyzed using ClustalW of the EMBL-EBI Job Dispatcher sequence analysis tools framework (https://www.ebi.ac.uk/jdispatcher). The obtained data were used to construct a phylogenetic tree using the iTOL v7 online tool^100^. Bootstrap analysis of the phylogenetic tree was executed using the IQ-TREE v2.3.6 tool, applying the substitution model with 1000 bootstrap replicates to evaluate branch length^101^. Further analyses were performed for potential signal-peptide signatures using SignalP (v6.0) (https://services.healthtech.dtu.dk/services/SignalP-6.0) with the default settings for eukaryotes. Subcellular localization analyses were conducted using TargetP (v1.1) and DeepLoc (v2.1) tools. If any signal peptide sequence was identified on the proteins, mature protein sequences without signal peptide sequences were subjected to subcellular localization analysis using the default settings for eukaryotes^102,103^. Identified secreted protein sequences were investigated for protein-protein interaction via String (v12.0) by setting the *Puccinia* and *Triticum* as the species of interest^88^. Proteins were further annotated for the presence of conserved domain structures using the CD Search tool for function prediction (https://www.ncbi.nlm.nih.gov/Structure/wrpsb-out/wrpsb.cgi). Pathogen host interaction database (PHI-Base) was used to annotate virulence attributions by BlastP search with a cut-off value smaller than 10^− 10 105^.

### HIGS analysis of the candidate effector PtVF1

Barley Stripe Mosaic Virus (BSMV) derived vectors were used for HIGS application. Constructions of the BSMV vectors (pα, pβΔβa, and pϒ) were done as described previously by Holzberg et al. ^106^. The coding sequence of PTTG_06852 was obtained from http://fungi.ensembl.org/index.html and aligned with the identified peptides in this study. The matching region was selected as the target of RNAi silencing, and PCR primers were designed. *Pac*I site and *Not*I site were added to the forward and reverse ends of the primer (Pt.06852-F: 5’-ATATTAATTAAGCTGGACTTGCCTTTGCTTTC-3’; Pt.06852-R: 5’-TATGCGGCCGC AATTCCTTGCCTCCGAAGTT-3’) for positional cloning into the pϒ component vector. The RNAi fragments were amplified using the designed primer pairs from cDNA derived from leaves 2 days after infection (dpi) and subcloned into the pJET1.2 vector. The ensuring plasmid pJET.06852 was isolated and cut by *Pac*I and *Not*I to generate the proper 5’ and 3’ ends, and this RNAi insert was then cloned into the BSMV pϒ vector. The insert was cloned into the pϒ vector separately in the sense (s) and antisense (as) orientation and transformed into the *E. coli*. Five clones for each were confirmed by PCR and Sanger sequencing.

The HIGS method was applied according to the work of Panwar and Bakkeren^57^. Seed from wheat cultivar ‘Morocco’, susceptible to *Pt*, was used for plant material. The seeds were germinated, and at the 2-leaf stage, seedlings were inoculated with BSMV to deliver the dsRNA to the plant cell. For this, pα, pβΔβa and pϒ.06852s, pϒ.06852as, pϒ.PDSs, pϒ.PDas, pϒ.0 (without insert) vectors were linearized by restriction enzymes MluI, PauI, and BssHII, respectively. Linearized plasmids were used as a template for the viral transcripts, which were transcribed using the T7 mMESSAGE mMACHINE kit (Ambion, AM1344). To reconstitute the complete tripartite BSMV genome (**α:βΔβa:ϒ**), an equal amount of transcripts (1 µg each) was combined and mixed in the inoculation solution (FES:77 mM glycine, 60 mM K_2_HPO_4_, 22 mM Na_4_P_2_O_7_·10H_2_O, 1% (w/v) bentonite, and 1% (w/v) Celite) and used for inoculation of the 2nd leaf. To silence the *PTTG_06852* gene, a mix of ϒ.06852s and ϒ.06852as in equal amounts was used (BSMV:06852, α:βΔβa:ϒ.06852s/as). As a control for BSMV effect on the *Pt* infection, ϒ.0, which does not contain any insert, was used (BSMV:0, α:βΔβa:ϒ.0). Additionally, ϒ.PDS, which expresses a *Phytoene desaturase* (PDS) gene fragment, was used for positive control (BSMV: PDS, α:βΔβa:ϒ.PDSs/as) of RNAi silencing, and a transcript-free FES solution was used for the mock inoculation. For each group, 10 seedlings were used, and 3 technical replicates were done. At 10 dpi, when BSMV symptoms appeared as white streaks on the leaves, the seedlings were inoculated with fresh *Pt* urediniospores. Samples for molecular and microscopic evaluation were collected from the 3rd and 4th leaves at 3 dpi and 5 dpi, respectively. At 12 dpi, the severity of *Pt* infection was assessed phenotypically on the remaining leaves after sampling by using a modified Cobb scale^107^. The hyphal lengths were recorded using 3 different urediniospores from 5 independent leaf samples (*n* = 15) for each group, namely, BSMV:0, BSMV:06852 (3rd leaves), and BSMV:06852 (4th leaves). The Kruskal-Wallis test was applied due to the non-normal distribution of the samples using IBM SPSS Statistics (Version 27).

Molecular evaluation of the suppression of *PTTG_06852* by HIGS was carried out by qRT-PCR. Total RNA was extracted from the samples using TRIzol Reagent (Invitrogen), and 1 µg of RNA was used for cDNA synthesis. *PTTG_06852* gene-specific primers (Pt.06852_qPCR_F:5’-TCAAAGTGGTCCTTGACACG-3’; Pt.06852_qPCR_R: 5’- TCGCAATCGTACTTGGAGTG − 3) and *Pt Succinate dehydrogenase* gene primers for internal control (Pt.sdh-qPCR-F: 5’-GGTTCCAGCGATAGATCGAG-3’; Pt.sdh-qPCR-R: 5’-CAACTACGACCAGCCACTCA-3’) were used for qRT-PCR analysis^108^. Changes in the developmental stages of the *Pt* urediniospores during infection were evaluated microscopically by the trypan blue staining method^109^. qRT-PCR was also performed to determine the fungal biomass by using *T. aestivum* and *Pt* housekeeping genes, *TaEF1 and PtRTP*, respectively^108^. The final evaluation of the infection severity was conducted according to the Cobb scale.

## Supplementary Information

Below is the link to the electronic supplementary material.

Supplementary Material 1

Supplementary Material 2

## Data Availability

The data is provided within the manuscript and supplementary information. The mass spectroscopy data is also deposited to the ProteomeXchange Consortium via the PRIDE partner repository with the dataset identifier PXD007162.
