# Supplementary material for: The proteome study of germinated Puccinia triticina urediniospores reveals a novel effector protein required for virulence
Source: Sci Rep. 2026 Apr 11;16:15726. doi: 10.1038/s41598-026-44996-2 (PMC13190833; doi:10.1038/s41598-026-44996-2)
Supplement: Supplementary file 2 — Supplementary Material 2 [file 41598_2026_44996_MOESM2_ESM.docx]

**Supplementary Information 1**

**The proteome study of germinated *Puccinia triticina* urediniospores reveals a novel effector protein required for virulence**

Ahmet Caglar Ozketen^1^, Merve Gecerer Cetinturk^2^, Christof Rampitsch^3^, Elifgul Aksu Tatlıses ^4^, M. Burak Tatlises^4^, Guus Bakkeren^5^, Semra Hasancebi^4^, Aslihan Gunel^2^*

^1^DESAM Research Institute, Near East University, Mersin 10, Türkiye

^2^Ahi Evran University, Faculty of Arts and Sciences, Department of Chemistry/Biochemistry, 40200 Kirsehir-Türkiye

^3^Morden Research and Development Centre, Agriculture and Agrifood Canada, 101 Route 100, Morden MB, R6M 1Y5, Canada

^4^Trakya University, Faculty of Engineering, Department of Genetics and Bioengineering, Edirne, 22100, Türkiye

^5^ Summerland Research and Development Centre, Agriculture and Agri-Food Canada, 4200 Hwy 97, Summerland, BC V0H 1Z0, Canada

*Corresponding Author: Dr. Aslihan Gunel

Email: [agunel@ahievran.edu.tr](mailto:agunel@ahievran.edu.tr), agunel@ahievran.edu.trgunel.aslihan@gmail.com

**Supplementary Information content:**

Supplementary Figures S1-S6

Supplementary Tables S1


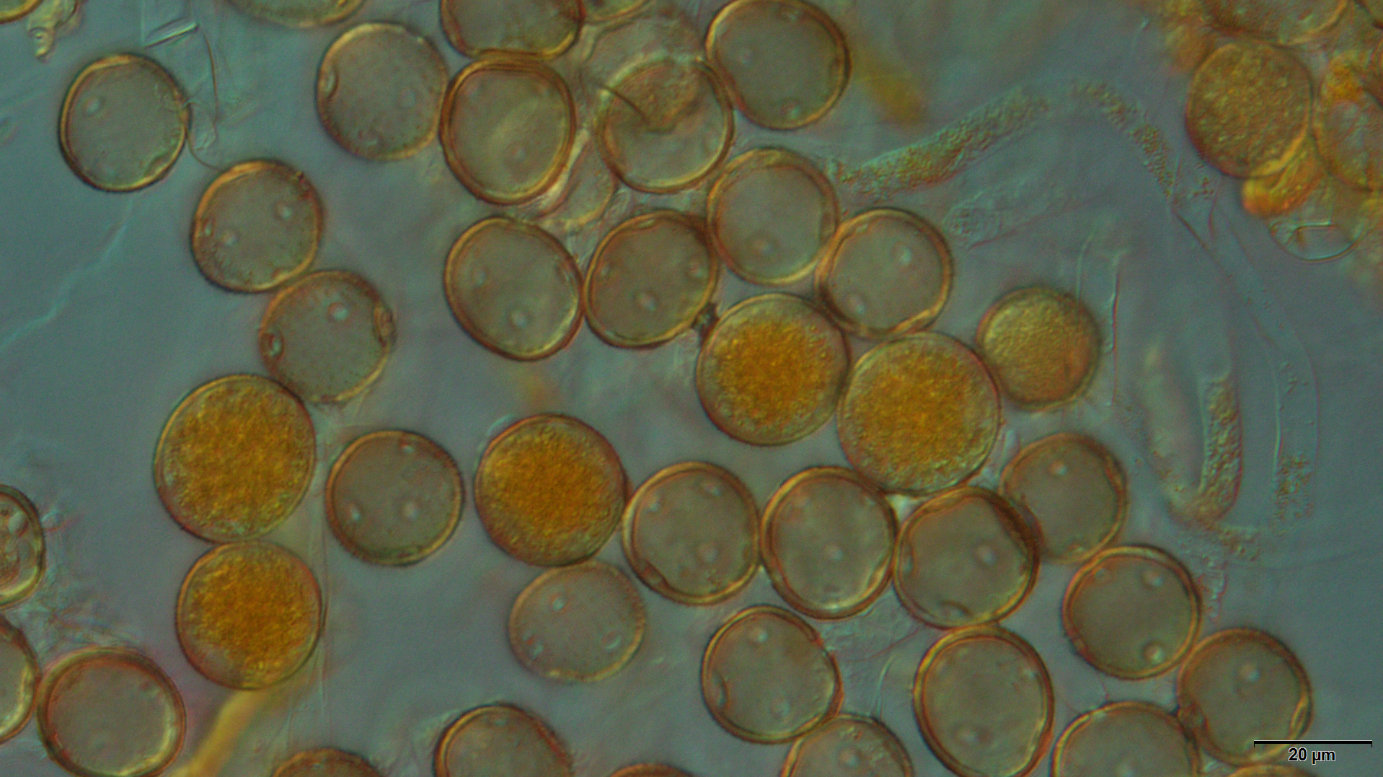


**Supplementary Figure S1.** Germinated urediniospores detailed photographs were captured by Olympus BX53 research microscope supplied with Olympus DP22 digi-CAM (Japan) and Axio imager 2 equipped with Nomarski differential interference contrast optics.


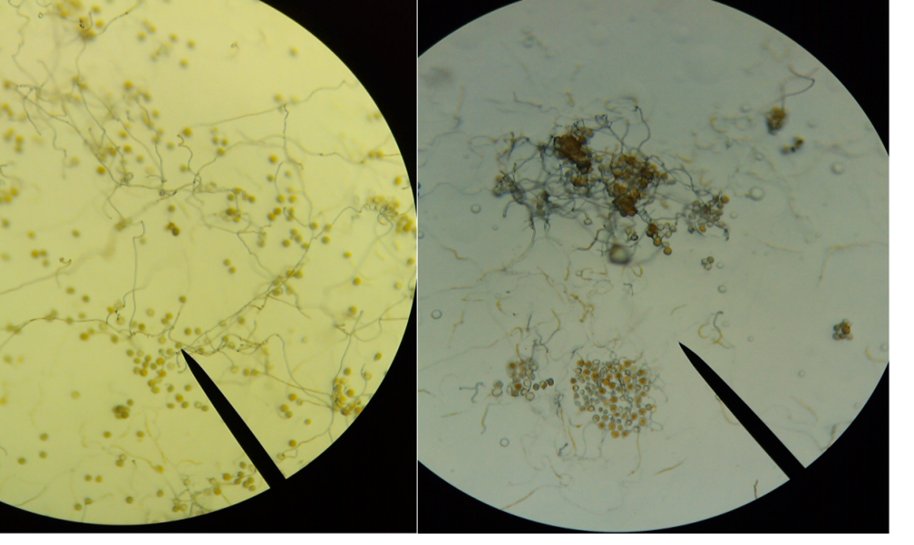


**Supplementary Figure S2.** Germinated uredinospores of Puccinia triticina race 1 on 0.5% (w/v) agarose in water: Leica DM 500 with 40X magnification. Germination was performed at 20 oC, in dark for 24 h in dew chamber. 3 mg uredinospores was distributed on agarose for each 5 mm petri dishes. Photograph was taken from both side of petri dishes since germ tubes are lying bottom of the petri dishes.


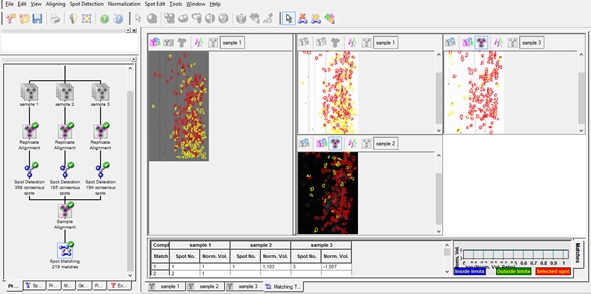


a

**Supplementary Figure S3.** Image analysis: All gels belonging to each biological replicates and their analytical replicates. Dymension (Syngene) 2D gel analysis software.


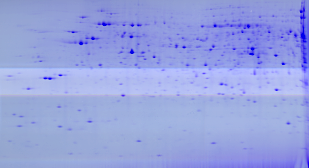

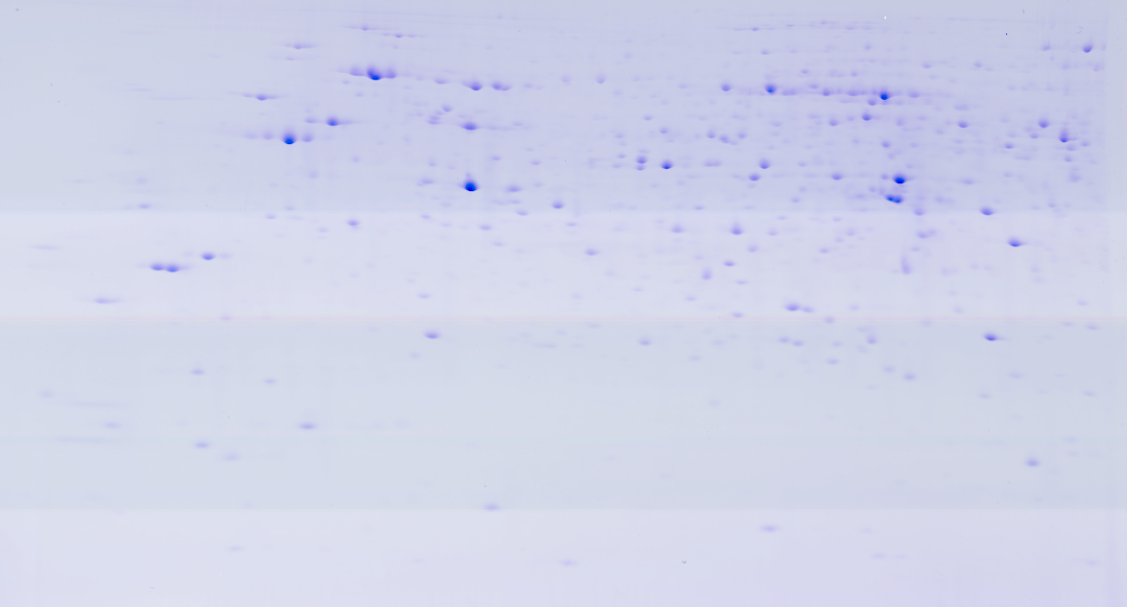

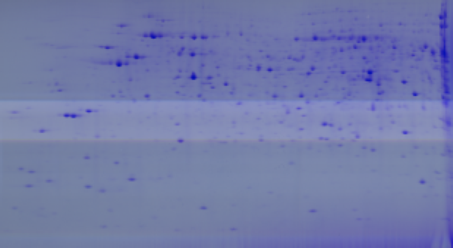


1-2

1-1

1-3


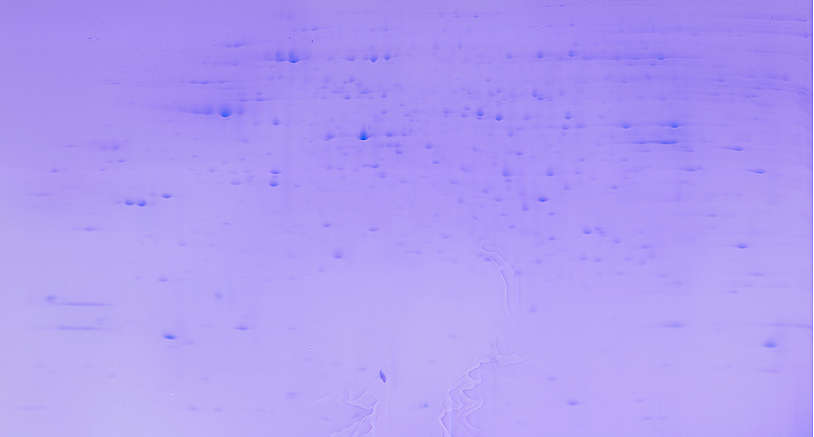

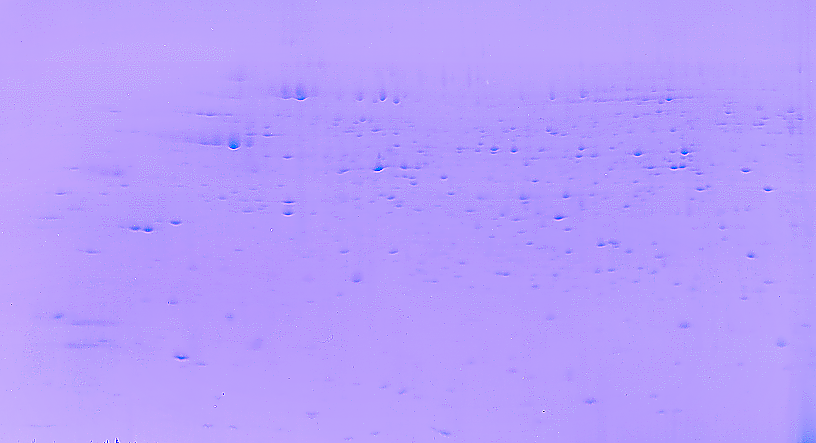

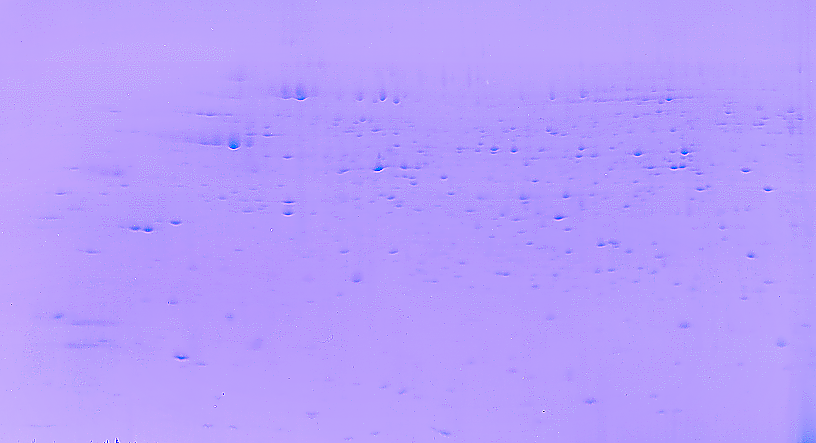


2-3

2-2

2-1


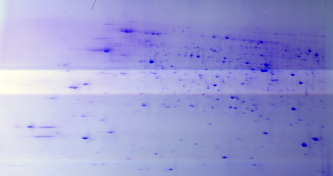

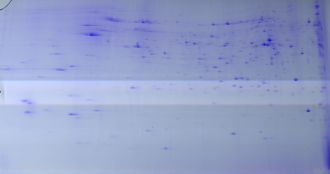

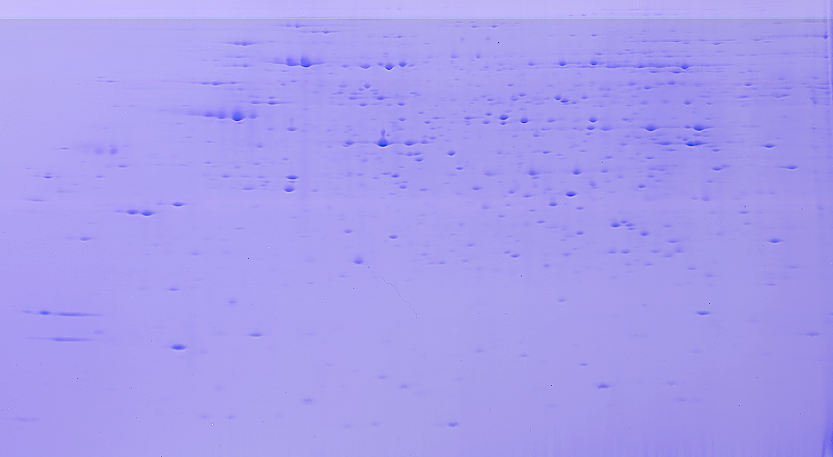


3-3

3-2

3-1

**Supplementary Figure S4.** Gel images of all biological replicates (3 biological replicates and 3 analytical replicates)


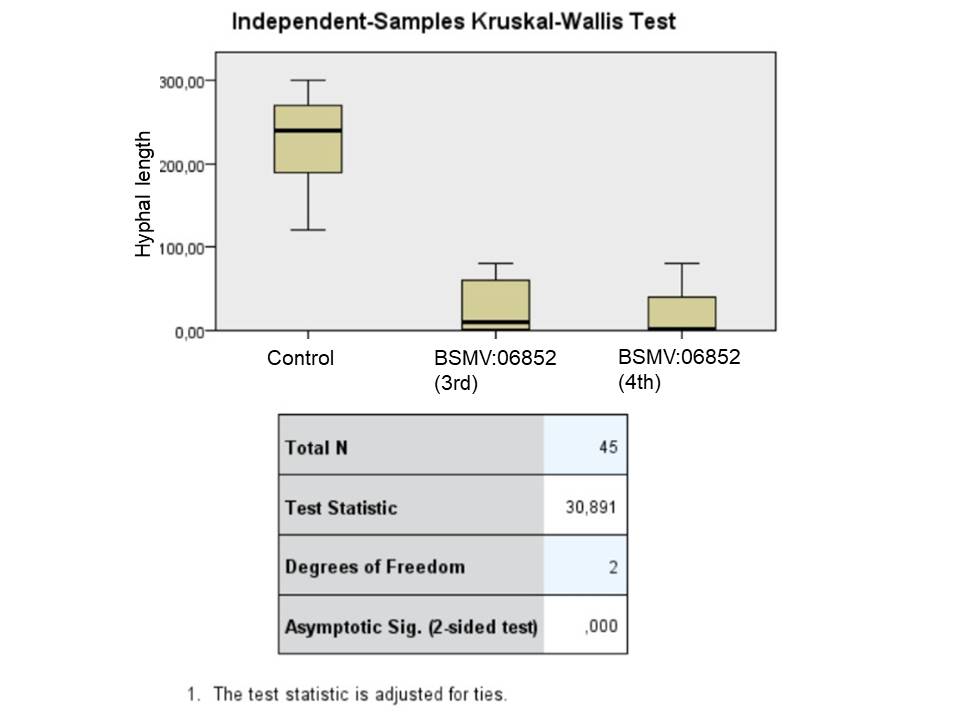


**Supplementary Figure S5.** The comparison of hyphae impairment associated with PtVF1 silencing. The box plot shows independent samples Kruskal-Wallis test results (p<0.001) generated by IBM SPSS Statistics (Version 27). Control group: BSMV:0 (n=15), BSMV:06852 (3^rd^ ): PtVF1 silenced 3^rd^ leaf of wheat (n=15), BSMV:06852 (4^th^): PtVF1 silenced 4^th^ leaf of wheat samples.


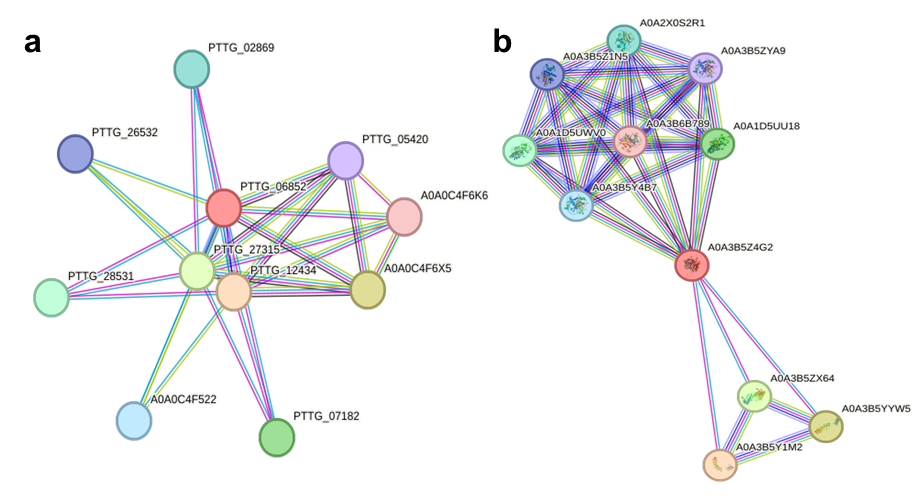


**Supplementary Figure S6.** Protein-protein interaction network of **(a)** PTTG_06852 and **(b)** its *Triticum aestivum* homolog A0A3B5Z4G2 (E-value: 2.7e-77). Analysis was carried out by STRING V.10 (<http://string-db.org)>. Nodes represent proteins and edges represent protein-protein association. Light blue and pink lines: known Interactions, Green, dark blue, and red lines: predicted interactions.

**Table S1**. List of identified proteins in germinated *P. triticina*

| \| Spot No \|  \|  \|  \|  \| \| --- \| --- \| --- \| --- \| --- \| | Exp pI/ Mw | Hits | Name | Theo pI/Mw |
| --- | --- | --- | --- | --- | --- | --- | --- | --- | --- |
| 3 | 4,33/35.21 | PTTG_03499 | Hypothetical protein | 4.45/24.82 |
| 7 | 4.42/55.87 | PTTG_01669 | Hypothetical protein | 4.71/26.90 |
|  |  | PSTG_04309 | Hypothetical protein | 5.13/47.07 |
| 12 | 4.59/26.33 | PTTG_09356 | Translationally-controlled tumor protein | 4.71/18.50 |
| 14 | 4.62/94.92 | PTTG_06852 | Hypothetical protein | 4.90/46.71 |
| 15 | 4.63/44.74 | PTTG_02090 | Hypothetical protein | 4.61/34.02 |
|  |  | PSTG_04309 | Hypothetical protein | 5.13/47.07 |
| 18 | 4.72/78.65 | PTTG_01840 | ATP synthase subunit beta, mitochondrial | 5.63/58.44 |
| 19 | 4.68/11.79 | PTTG_05456 | Hypothetical protein | 5.17/13.16 |
| 20 | 4.63/94.92 | PTTG_01827 | Hypothetical protein | 4.86/58.40 |
| 21 | 4.71/78.65 | PTTG_01840 | ATP synthase subunit beta, mitochondrial | 5.63/58.44 |
| 22 | 4.72/54.92 | PTTG_07476 | Pyruvate dh e1 component | 5.55/43.09 |
|  |  | PTTG_02217 | Hypothetical protein | 4.97/35.48 |
| 23 | 4.79/124.78 | PTTG_06867 | Heat shock 90-1 | 4.89/80.59 |
| 24 | 4.72/94.92 | PTTG_01827 | Hypothetical protein | 4.86/58.40 |
| 27 | 4.75/25.67 | PTTG_27503 | Hypothetical protein | 4.99/18.87 |
| 30 | 4.76/124.78 | PTTG_06867 | HSP 90-1 | 4.89/80.59 |
| 31 | 4.78/54.92 | PTTG_02217 | Hypothetical protein | 4.97/35.48 |
|  |  | PTTG_07476 | Pyruvate dh e1 component | 5.55/43.09 |
| 35 | 4.8/87.14 | PGTG_12204 | Tubulin beta chain | 4.82/50.15 |
| 38 | 4.82/21.27 | PTTG_04893 | Eukaryotic translation initiation factor 5a(elongation factor p) | 5.14/17.31 |
| 39 | 4.83/53.08 | PTTG_09144 | Hypothetical protein) | 5.41/57.84 |
| 41 | 4.84/87.14 | PGTG_12204 | Tubulin β chain | 4.82/50.15 |
| 42 | 4.85/112.61 | PTTG_03478 | HSP-70 | 5.09/70.90 |
| 46 | 4.87/87.14 | PGTG_12204 | Tubulin β chain | 4.82/50.15 |
|  |  | PTTG_05513 | 26S protease regulatory subunit 6a | 5.09/51.99 |
| 48 | 4.88/110.70 | PTTG_07281 | Glucose regulated protein | 5.16/72.28 |
| 50 | 4.9/54.92 | PTTG_07437 | Hypothetical Protein | 5.55/78.72 |
| 51 | 4.9/74.71 | PTTG_01758 | Hypothetical Protein | 5.06/31.69 |
|  |  | PTTG_03478 | HSP-70 | 5.09/70.90 |
| 53 | 4.92/110.70 | PTTG_07281 | Glucose regulated protein | 5.16/72.28 |
|  |  | PTTG_06867 | HSP 90-1 | 4.89/80.59 |
| 55 | 4.95/110.70 | PTTG_07281 | Glucose regulated protein | 5.16/72.28 |
| 58 | 4.99/110.70 | PTTG_07281 | Glucose regulated protein | 5.16/72.28 |
|  |  | PTTG_26408 | V-type ATPase, a subunit | 5.91/76.81 |
| 63 | 5.02/96.55 | PTTG_03478 | HSP70 | 5.09/70.90 |
| 64 | 5.03/47.09 | PTTG_03718 | Hypothetical Protein | 5.10/28.50 |
|  |  | PTTG_01641 | Hypothetical Protein | 5.14/31.97 |
| 65 | 5.06/30.19 | Contaminant |  |  |
| 66 | 5.06/68.59 | PSTG_03501 | Hypothetical Protein | 5.31/41.78 |
| 67 | 5.06/57.81 | PSTG_03501 | Hypothetical Protein | 5.31/41.78 |
| 70 | 5.07/108.83 | PTTG_26408 | V-type ATPase, a subunit | 5.91/76.81 |
| 73 | 5.08/96.55 | PTTG_05827 | HSP60 | 5.64/61.84 |
| 73A | 5.07/90.17 | PTTG_01106 | Tubulin ∝1 chain | 5.09/49.06 |
| 74 | 5.09/33.45 | PTTG_03524 | Hypothetical Protein | 6.10/28.28 |
| 75 | 5.09/62.97 | PTTG_06777 | Thioredoxin reductase (NADPH) | 5.58/41.87 |
| 76 | 5.1/56.83 | PTTG_09204 | Hypothetical Protein | 5.06/48.96 |
| 78 | 5.12/94.92 | PTTG_09204 | Hypothetical protein | 5.06/48.96 |
| 79 | 5.14/90.17 | PTTG_01106 | Tubulin ∝1 chain | 5.09/49.06 |
|  |  | PTTG_03808 | Protein phosphatase 2c | 5.17/54.31 |
| 80 | 5.14/108.83 | PTTG_01696 | HSP SSB | 5.34/68.34 |
| 82 | 5.16/68.59 | PSTG_03501 | Actin | 5.31/41.78 |
|  |  | PGTG_05935 | HSP SSB | 5.34/69.20 |
| 84 | 5.19/76.02 | PSTG_03501 | Actin | 5.31/41.78 |
| 87 | 5.17/90.17 | PSTG_03501 | Actin | 5.31/41.79 |
| 88 | 5.19/56.83 | PTTG_12630 | Arginase | 5.25/34.78 |
| 90 | 5.2/66.29 | PSTG_12276 | Hypothetical protein | 5.17/54.31 |
| 91 | 5.2/78.65 | PTTG_00226 | 26S protease regulatory subunit 6b | 5.33/46.73 |
| 92 | 5.21/91.73 | PTTG_09144 | Hypothetical protein | 5.41/57.84 |
| 93 | 5.21/53.08 | PTTG_06345 | Hypothetical protein | 5.29/43.80 |
| 95 | 5.24/69.78 | PTTG_09276 | Hypothetical protein | 5.77/80.76 |
| 96 | 5.24/93.31 | PTTG_09144 | Hypothetical protein | 5.41/57.84 |
| 97 | 5.24/65.16 | PTTG_08592 | 3-isopropyl malate dehydrogenase | 5.39/39.88 |
|  |  | PGTG_13966 | Hypothetical protein | 6.97/48.13 |
| 98 | 5.24/110.70 | PTTG_26408 | V-type ATPase, a subunit | 5.91/76.81 |
| 99 | 5.26/61.91 | PSTG_14021 | V-type proton ATPase catalytic subunit A isoform 2 | 5.44/68.91 |
| 100 | 5.27/110.70 | PGTG_05935 | HSP-SSB | 5.34/68.95 |
| 101 | 5.28/77.31 | PTTG_02219 | Phosphomannose isomerase | 5.44/48.00 |
| 104 | 5.33/114.55 | PTTG_01076 | Hsp-70 like protein | 5.88/73.63 |
| 105 | 5.33/65.16 | PSTG_04289 | Hypothetical protein | 6.62/49.00 |
|  |  | PTTG_09276 | Hypothetical protein | 5.77/80.76 |
| 108 | 5.36/58.81 | PTTG_03022 | Hypothetical protein | 5.00/29.33 |
| 111 | 5.38/42.50 | PTTG_09887 | Hypothetical protein | 5.64/29.99 |
|  |  | PSTG_02641 | CMGC/CDK/CDC2 protein kinase | 6.26/33.15 |
|  |  | PTTG_01991 | Hypothetical protein | 5.40/32.48 |
| 112 | 5.41/114.55 | PTTG_01076 | HSP-70 | 5.88/73.63 |
| 114 | 5.41/12.20 | PTTG_09971 | Hypothetical protein | 5.86/14.07 |
| 115 | 5.38/39.69 | PGTG_00252 | Hypothetical protein | 5.49/12.32 |
| 116 | 5.42/52.18 | PTTG_03284 | LSU ribosomal protein | 5.25/33.28 |
|  |  | PSTG_01609 | Serine/threonine-protein phosphatase ppe1 | 5.43/34.92 |
| 119 | 5.44/106.98 | PTTG_09796 | Hypothetical protein | 5.47/65.89 |
| 121 | 5.45/77.31 | PTTG_04091 | Secretory pathway GDP dissociation inhibitor1 | 5.64/49.56 |
| 122 | 5.46/47.09 | PTTG_09784 | Hypothetical protein | 5.95/69.87 |
|  |  | PTTG_03046 | PEP carboxy kinase | 6.34/64.32 |
|  |  | PTTG_06246 | Hypothetical protein | 5.87/30.68 |
| 126 | 5.48/57.81 | PTTG_01458 | PEP carboxy kinase | 6.23/64.25 |
| 127 | 5.49/116.53 | PTTG_01076 | HSP-70 like protein | 5.88/73.63 |
|  |  | PTTG_09276 | Hypothetical protein | 5.77/80.76 |
| 128 | 5.5/64.06 | PTTG_09870 | Hypothetical protein | 5.72/41.37 |
| 130 | 5.5/81.38 | PTTG_01076 | HSP-70 like protein | 5.88/73.63 |
| 133 | 5.52/39.69 | PTTG_02001 | Hypothetical protein | 8.30/59.21 |
|  |  | PTTG_07863 | Hypothetical protein | 8.30/20.75 |
| 136 | 5.54/34.62 | PTTG_07672 | Proteasome subunit alpha type-2 | 5.71/26.89 |
| 137 | 5.55/60.86 | PTTG_07672 | Proteasome subunit alpha type-2 | 5.71/26.89 |
| 139 | 5.55/91.73 | PTTG_08656 | Hypothetical protein | 5.63/41.41 |
|  |  | PTTG_08249 | Hypothetical protein | 5.62/56.64 |
| 140 | 5.56/78.65 | PTTG_08249 | Hypothetical protein | 5.62/56.64 |
| 141 | 5.56/52.18 | PTTG_07672 | Proteasome subunit alpha type-2 | 5.71/26.89 |
|  |  | PTTG_08249 | Hypothetical protein | 5.62/56.64 |
| 142 | 5.56/88.64 | PTTG_07672 | Proteasome subunit alpha type-2 | 5.71/26.89 |
| 144 | 5.59/66.29 | PTTG_07672 | Proteasome subunit alpha type-2 | 5.71/26.89 |
| 146 | 5.70/66.29 | PTTG_00430 | ENOLASE | 5.97/47.38 |
| 147 | 5.60/58.81 | PTTG_07672 | Proteasome subunit alpha type-2 | 5.71/26.89 |
| 149 | 5.62/81.38 | PTTG_06888 | (Haloacid dehalogenase-like hydrolase DOM.) | 5.58/38.67 |
|  |  | PTTG_02620 | Mitochondrial-processing peptidase subunit beta | 6.03/52.87 |
| 150 | 5.62/74.71 | PTTG_01903 | Hypothetical protein | 6.17/46.40 |
| 154 | 5.64/52.18 | PTTG_01903 | Hypothetical protein | 6.17/46.40 |
| 158 | 5.66/48.73 | PTTG_07672 | Proteasome subunit alpha type-2 | 26.89/5.71 |
| 160 | 5.68/114.55 | PTTG_09276 | Hypothetical protein | 5.77/80.76 |
| 161 | 5.68/90.17 | PTTG_06273 | Hypothetical protein | 5.82/49.91 |
| 163 | 5.69/59.82 | PTTG_07362 | V-type proton ATPase subunit b | 5.66/57.11 |
| 164 | 5.69/26.33 | PGTG_01136 | Fructosebiphosphate aldolase class2 | 39,43/5,60 |
| 165 | 5.70/35.21 | PTTG_01903 | Hypothetical protein | 6.17/46.40 |
| 167 | 5.70/88.64 | PTTG_09553 | Hypothetical protein | 5.67/5137 |
|  |  | PTTG_28577 | Hypothetical protein | 6.34/56.48 |
|  |  | PTTG_09276 | Hypothetical protein | 5.77/80.76 |
| 169 | 5.72/51.29 | PTTG_03429 | Hypothetical protein | 6.39/37.37 |
| 170 | 5.72/91.73 | PTTG_00372 | Glutathione synthetase | 5.77/58.90 |
| 171 | 5.72/61.91 | PTTG_09731 | Hypothetical protein | 8.79/57.52 |
| 172 | 5.73/59.83 | PTTG_05189 | Fructose biphosphate aldolase class ii | 6.83/46.24 |
| 174 | 5.74/36.44 | PTTG_09731 | Hypothetical protein | 8.79/57.52 |
| 175 | 5.72/112.61 | PTTG_09276 | Hypothetical protein | 5.77/80.76 |
| 176 | 5.74/40.38 | PTTG_07572 | Phospho mannomutase | 5.84/29.12 |
| 178 | 5.75/47.09 | PTTG_01458 | PEP carboxy kinase | 6.23/64.25 |
| 181 | 5.76/76.00 | PTTG_03422 | 6-PHospho gluconate dehydrogenase | 6.77/68.75 |
| 182 | 5.76/54.92 | PTTG_04265 | Hypothetical protein | 7.78/36.47 |
| 184 | 5.77/81.38 | PTTG_03422 | 6-phospho gluconate dehydrogenase | 6.77/68.75 |
| 185 | 5.77/114.55 | PTTG_09276 | Hypothetical protein | 5.77/80.76 |
| 186 | 5.79/96.55 | PTTG_04566 | Hypothetical protein | 5.71/56.71 |
| 187 | 5.79/58.81 | PTTG_08360 | Pyruvate kinase | 6.43/57.63 |
| 188 | 5.82/15.50 | PTTG_01714 | Fk506 binding protein | 6.57/12.88 |
| 190 | 5.8/77.31 | PTTG_09784 | Hypothetical protein) | 5.95/69.87 |
| 191 | 5.82/90.17 | PTTG_00430 | Enolase | 5.98/47.25 |
| 192 | 5.82/112.61 | PTTG_09276 | Hypothetical protein | 5.77/80.76 |
| 193 | 5.82/64.06 | PSTG_11939 | Hypothetical protein | 6.77/267.70 |
|  |  | PGTG_21560 | Hypothetical protein | 8.69/78.72 |
| 194 | 5.84/36.44 | PTTG_03025 | Hypothetical protein | 5.86/21.37 |
|  |  | PTTG_07306 | 2,3-bisphosphoglycerate-dependent phosphoglycerate mutase | 5.88/23.82 |
| 195 | 5.85/69.78 | PTTG_08248 | Hypothetical protein | 7.5945.24 |
| 197 | 5.85/42.50 | PTTG_09038 | Hypothetical protein | 6.00/28.96 |
| 198 | 5.85/112.61 | PTTG_09038 | Hypothetical protein | 6.00/28.96 |
| 199 | 5.86/81.38 | PTTG_09038 | Hypothetical protein | 6.00/28.96 |
| 200 | 5.86/73.45 | PTTG_03445 | Hypothetical protein | 6.20/22.59 |
| 201 | 5.87/101.64 | PTTG_01903 | Hypothetical protein | 6.17/46.40 |
|  |  | PSTG_11939 | Hypothetical protein | 6.77/267.79 |
| 202 | 5.87/118.54 | PTTG_09276 | Hypothetical protein | 5.77/80.76 |
| 204 | 5.87/35.82 | PTTG_07306 | 2,3-bisphosphoglycerate-dependent phosphoglycerate mutase | 5.88/23.82 |
| 209 | 5.89/42.50 | PTTG_27324 | Hypothetical protein | 5.90/29.41 |
| 210 | 5.9/82.79 | PTTG_01903 | Hypothetical protein | 6.17/46.40 |
| 212 | 5.91/1112.61 | PTTG_09276 | Hypothetical protein | 5.77/80.76 |
|  |  | PTTG_09784 | Hypothetical protein | 5.95/69.87 |
| 214 | 5.91//66.29 | PTTG_03594 | CMGC/MAPK protein kinase | 39,70/5,91 |
| 216 | 5.91/90.17 | PTTG_29605 | Hypothetical protein | 7.23/58.85 |
| 218 | 5.92/99.91 | PTTG_08360 | Pyruvate kinase | 6.43/57.63 |
| 220 | 5.94/33.46 | PTTG_03445 | Hypothetical protein | 6.20/22.59 |
|  |  | PSTG_01145 | Hypothetical protein | 5.70/37.88 |
| 221 | 5.93/114.55 | PTTF_09276 | Hypothetical protein | 5.77/80.76 |
|  |  | PSTG_08642 | Hypothetical protein | 6.10/125.69 |
| 222 | 5.94/40.37 | PTTG_08360 | Pyruvate kinase | 6.43/57.63 |
| 223 | 5.93/77.31 | PTTG_08360 | Pyruvate kinase | 6.43/57.63 |
| 224 | 5.99/64.06 | PTTG_08772 | Hypothetical protein | 6.24/41.04 |
| 225 | 5.96/110.70 | PTTG_05785 | Succinate dehydrogenase [ubiquinone] flavoprotein subunit, mitochondrial | 6.67/70.79 |
| 227 | 5.95/36.44 | PTTG_01458 | PEP carboxykinase | 6.23/64.25 |
| 231 | 5.97/114.55 | PTTG_05785 | Succinate dehydrogenase [ubiquinone] flavoprotein subunit, mitochondrial | 6.67/70.79 |
| 234 | 5.97/91.73 | PTTG_01458 | PEP carboxykinase | 6.23/64.25 |
| 257 | 6.04/116.53 | PTTG_09784 | Hypothetical protein | 5.95/69.87 |
| 261 | 6.05/93.31 | PTTG_00016 | Hypothetical protein | 6.25/58.93 |
|  |  | PTTG_12239 | Hypothetical protein | 7.10/97.98 |
| 263 | 6.06/88.64 | PTTG_12239 | Hypothetical protein | 7.10/97.98 |
| 267 | 6.08/72.20 | PTTG_05453 | Glutamine synthetase | 6.15/39. 60 |
|  |  | PTTG_08909 | CaMK protein kinase | 6.03/40.32 |
| 269 | 6.08/80.00 | PTTG_00430 | Enolase | 5.97/47.38 |
| 272 | 6.1/118.54 | PTTG_09784 | Hypothetical protein | 5.95/69.87 |
| 274 | 6.1/84.21 | PTTG_01684 | Hypothetical protein | 8.53/50.22 |
| 281 | 6.13/135.91 | PTTG_03798 | NADH dehydrogenase g-subunit | 6.62/81.62 |
| 285 | 6.13/69.78 | PTTG_06073 | Hypothetical protein | 8.05/47.74 |
| 286 | 6.13/76.00 | PTTG_03821  PTTG_04794 | Adenosyl homocysteinase | 5.97/47.06 |
|  |  |  | Elongation factor EF-Tu | 8.53/52.40 |
| 287 | 6.13/70.98 | PTTG_06073 | Hypothetical protein | 8.05/47.74 |
|  |  | PTTG_06742 | Hypothetical protein | 5.83/102.56 |
| 290 | 6.13/118.54 | PTTG_05785 | Succinate dehydrogenase | 6.67/70.79 |
| 294 | 6.16/41.78 | PTTG_01458 | PEP carboxy kinase | 6.23/64.25 |
| 296 | 6.17/73.45 | PTTG_01458 | PEP carboxy kinase | 6.23/64.25 |
| 299 | 6.19/53.08 | PTTG_04303 | Peptidyl-prolyl cis-trans isomerase | 6.54/26.53 |
| 305 | 6.2/105.17 | PTTG_09276 | Hypothetical protein) | 5.77/80.76 |
| 306 | 6.21/48.73 | PTTG_02168 | Phosphogluco mutase | 6.25/60.70 |
| 307 | 6.2/54.92 | PTTG_02168 | Phosphogluco mutase | 6.25/60.70 |
| 309 | 6.21/45.51 | PTTG_01152 | Hypothetical protein | 6.44/57.04 |
| 310 | 6.22/140.64 | PTTG_29181 | Hypothetical protein | 6.23/83.85 |
| 315 | 6.25/53.99 | PTTG_02169 | Hypothetical protein | 6.30/32.84 |
| 318 | 6.27/72.20 | PTTG_09428 | Hypothetical protein | 6.21/38.48 |
| 320 | 6.28/54.92 | PTTG_00389 | Guanine nucleotide-binding protein subunit beta-like protein | 6.31/34.73 |
| 321 | 6.28/30.71 | PTTG_05810 | 20S proteasome subunit beta | 5.94/23.71 |
| 322 | 6.3/76.00 | PTTG_01421 | Hypothetical protein | 6.39/44.49 |
| 332 | 6.33/62.97 | PTTG_29878 | Transaldolase | 6.30/38.78 |
| 337 | 6.35/103.39 | PTTG_01152 | Hypothetical protein | 6.44/57.04 |
| 338 | 6.36/34.03 | PTTG_06268 | Hypothetical protein | 6.22/23.14 |
| 340 | 6.36/108.83 | PTTG_06466 | Isocitrate lyase | 6.94/63.84 |
| 341 | 6.34/54.92 | PTTG_06754 | Catalase | 6.26/59.54 |
| 347 | 6.41/22.97 | PTTG_06754 | Catalase | 6.26/59.54 |
| 348 | 6.44/62.97 | PGTG_12204 | Tubulin β chain | 4.83/50.14 |
| 351 | 6.41/101.63 | PTTG_03356 | Glucose-6-phosphate isomerase | 6.47/61.41  71.16/8.98 |
|  |  | PTTG_04108 | Hypothetical protein |  |
| 356 | 6.45/138.26 | PGTG_17649 | UTP-glucose-1-phosphate uridylyl transferase | 6.25/57.18 |
